# Supplementary material for: Human HspB1, HspB3, HspB5 and HspB8: Shaping these disease factors during vertebrate evolution
Source: Cell Stress Chaperones. 2022 Jun 9;27(4):309–23. doi: 10.1007/s12192-022-01268-y (PMC9346038; doi:10.1007/s12192-022-01268-y)
Supplement: Supplementary file 1 — (PDF 112 KB) [file 12192_2022_1268_MOESM1_ESM.pdf]

Online Supplemental Materials
Figure S1

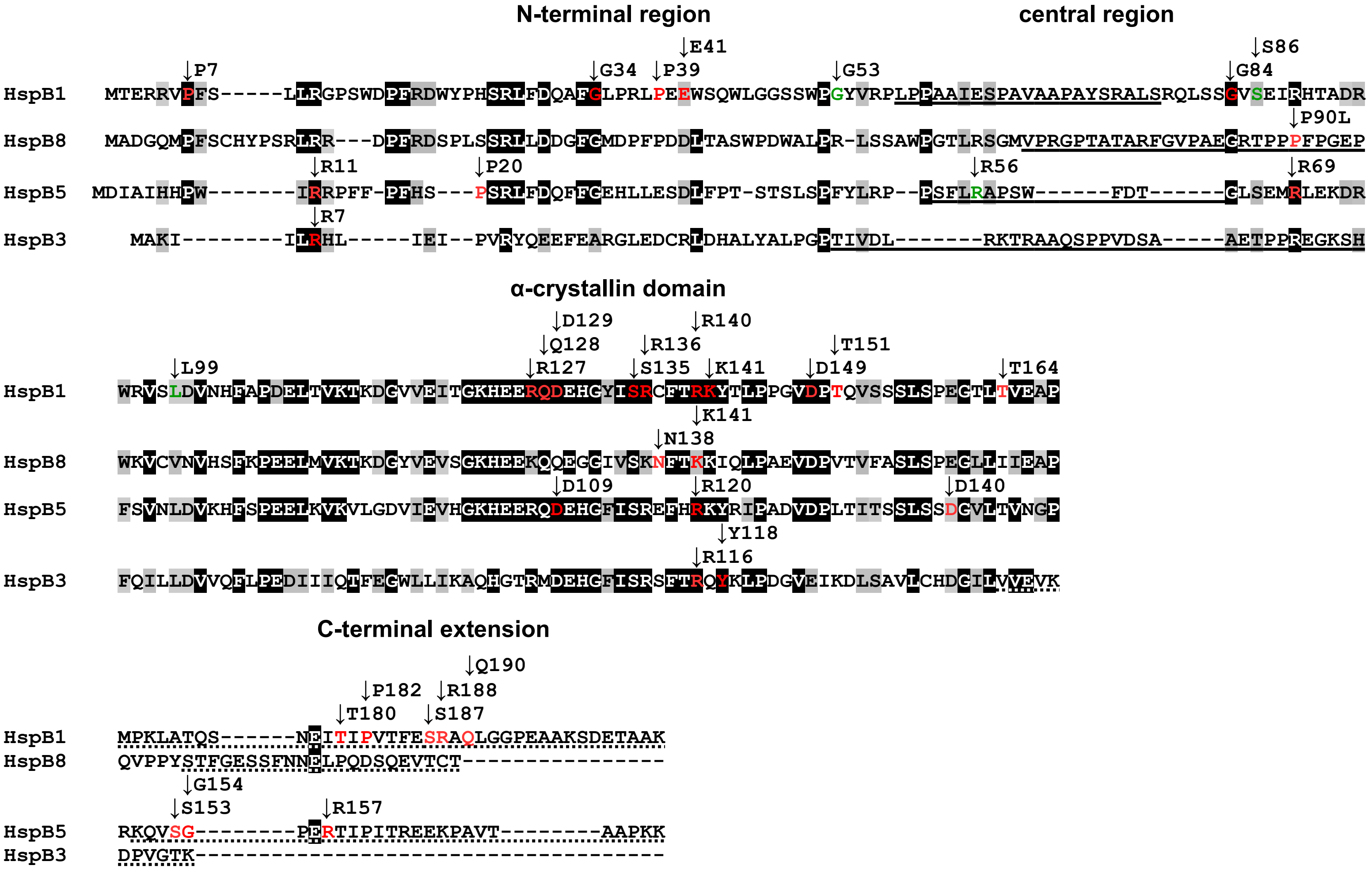

Domains and regions in the aligned human sequences of HspB1, HspB3, HspB5 and HspB8, and positions of the disease-associated missense mutations. The alignment was according to Fontaine et al. (2003) with minor modifications. Identical amino acid residues are highlighted in black if they occur in at least three sequences. Similar amino acid residues (E/D; A/G; H/F/W/Y; S/T; I/L/V; H/R/K) are highlighted in gray if they occur in at least three sequences, or in addition to two or three identical amino acid residues. Amino acid residues affected by missense mutations associated with various forms of neuropathy, myopathy, or with cataracts in the eye lens, are marked with color: red, mutation sites associated with a dominant disease phenotype (or this can be assumed); green, mutation sites associated with a recessive disease phenotype. Sequence sections with relatively high average dN/dS point estimates (ω-values; cf., Fig. 1), compared to the α-crystallin domain and the N-terminal region, are underlined (solid and dotted underline) and correspond approximately to the central regions and the C-terminal extensions, respectively. This demarcation served to define the sequence partitions as used in this study.
