# Supplementary material for: Human HspB1, HspB3, HspB5 and HspB8: Shaping these disease factors during vertebrate evolution
Source: Cell Stress Chaperones. 2022 Jun 9;27(4):309–23. doi: 10.1007/s12192-022-01268-y (PMC9346038; doi:10.1007/s12192-022-01268-y)
Supplement: Supplementary file 2 — (PDF 294 KB) [file 12192_2022_1268_MOESM2_ESM.pdf]

Online Supplemental Materials

Table S1. Reported disease-associated missense mutations in human HspB1, HspB3, HspB5 and HspB8[a]

sHSP	mutation (protein)	mutation (cDNA)	associated disease phenotype		references[f]
			symptoms	inheritance[b]	
HspB1	p.P7S	c.19C>T	dHMN	D	Echaniz-Laguna et al. 2017
	p.P7R	c.20C>G	CMT	D	Fortunato et al. 2017
	p.G34R	c.100G>A	dHMN	I (D)	Capponi et al. 2011
	p.P39L	c.116C>T	dHMN, CMT	D, S	Houlden et al. 2008; Capponi et al. 2011; Echaniz-Laguna et al. 2017; Rossor et al. 2017
	p.E41K	c.121G>A	dHMN	D	Capponi et al. 2011
	p.G53D	c.158G>A	dHMN + cerebrellar ataxia	R	Echaniz-Laguna et al. 2017
	p.G84R	c.250G>C c.250G>A	dHMN, CMT	D, S	Houlden et al. 2008; James et al. 2008, Rossor et al. 2017; Ho et al. 2017
	p.S86L	c.257C>T	dHMN + ALS-like	R	Scarlato et al. 2015
	p.L99M	c.295C>A	dHMN	R	Houlden et al. 2008; Rossor et al. 2017
	p.R127W	c.379C>T	dHMN, CMT, FP, LLS, ALS	D	Evgrafov et al. 2004; Tang et al. 2005a; Dierick et al. 2008; Echaniz-Laguna et al. 2017; Benedetti et al. 2010; Chen et al. 2021
	p.R127L	c.380G>T	CMT	D	Ylikallio et al. 2014
	p.Q128R	c.383A>G	dHMN, LLS	D	Echaniz-Laguna et al. 2017
	p.D129E	c.387C>G	dHMN + myopathy	D	Lewis-Smith et al. 2016
	p.S135F	c.404C>T	dHMN, CMT+FP	D, S	Evgrafov et al. 2004; Houlden et al. 2008; Chung et al. 2008; Echaniz-Laguna et al. 2017; Rossor et al. 2017
	p.S135C	c.404C>G	dHMN, CMT	D	Benedetti et al. 2010; Oberstadt et al. 2016
	p.S135Y	c.404C>A	CMT	D, S	Ylikallio et al. 2014; Rossor et al. 2017
	p.R136W	c.406C>T	CMT	D[c]	Evgrafov et al. 2004
	p.R136L	c.407G>T	dHMN, CMT + deafness + pyramidal signs	D, I	Capponi et al. 2011; Gaeta et al. 2012; Stancanelli et al. 2015
	p.R136H	c.407G>C	dHMN	(D)	Frasquet et al. 2021
	p.R140G	c.418C>G	dHMN, DM	S, SD[d]	Houlden et al. 2008; Rossor et al. 2017; Bugiardini et al. 2017
	p.K141Q	c.421A>C	dHMN	D	Ikeda et al. 2009; Maeda et al. 2014
	p.D149A	c.446A>C	ALS	(D)	Chen et al. 2021
	p.T151I	c.452C>T	dHMN	D	Evgrafov et al. 2004; Dierick et al. 2008; Echaniz-Laguna et al. 2017
	p.T151P	c.451A>C	ALS	(D)	Chen et al. 2021
	p.T164A	c.490A>G	CMT	D	Lin et al. 2011
	p.T180I	c.539C>T	dHMN, CMT	D, S	Luigetti et al. 2010; Capponi et al. 2011; Echaniz-Laguna et al. 2017
	p.P182L	c.545C>T	dHMN	PM	Evgrafov et al. 2004; Dierick et al. 2008
	p.P182S	c.544C>T	dHMN	S (D)	Kijima et al. 2005
	p.P182A	N/D	dHMN	D	Rossor et al. 2017
	p.S187L	c.560C>T	dHMN	S	Echaniz-Laguna et al. 2017

	p.R188W	c.562C>T	CMT	I (D)	Capponi et al. 2011
	p.Q190H	c.570C>G	ALS + cognitive impairment	S	Capponi et al. 2016
HspB3[e]	p.R7S	c.21G>T	dHMN	D	Kolb et al. 2010; Laššuthová et al. 2016
	p.R116P	c.347G>C	myopathy + axonal neuropathy	D	Morelli et al. 2017
	p.Y118H	c.352C>T	CMT	D	Nam et al. 2018
HspB5	p.P20S	c.58C>T	CC	D	Liu et al. 2006a
	p.R56W	c.166C>T	JC	R	Safieh et al. 2009
	p.D109H	c.325G>C	MFM + DCM + C	D	Sacconi et al. 2012
	p.D109G	c.326A>G	AxM + RCM	D	Brodehl et al. 2017
	p.R120G	c.358A>G	MFM + HCM+C	D	Vicart et al. 1998
	p.D140N	c.418G>A	CC	D	Liu et al. 2006b
	p.S153F	c.458>T	myopathy + HCM + C + cerebrellar ataxia + optic atrophy + cognitive impairment	(D)	Sadeh et al. 2021
	p.G154S	c.460G>A	DM + DCM	(D)	Pilotto et al. 2006; Reilich et al. 2010
	p.R157H	c.470G>A	DCM	(D)	Inagaki et al. 2006
HspB8	p.P90L	c.413A>C	dHMN	S	Echaniz-Laguna et al. 2017
	p.N138T	c.413A>C	dHMN	D	Echaniz-Laguna et al. 2017
	p.K141E	c.421A>G	dHMN, distal myopathy	D	Irobi et al. 2004; Dierick et al. 2008; Ghaoui et al. 2016
	p.K141N	c.423G>C	dHMN, CMT	D	Irobi et al. 2004; Dierick et al. 2008; Echaniz-Laguna et al. 2017
		c.423G>T	CMT	D	Tang et al. 2005b
	p.K141T	c.422A>C	CMT	S (D)	Nakhro et al. 2013
	p.K141M	c.422A>T	dHMN + FP	D	Echaniz-Laguna et al. 2017

[a] Abbreviations and symbols. Inheritance of phenotype: D, dominant; R, recessive; SD, semi-dominant; I, isolated (positive family history referred, parents not examined); S, sporadic (parents not affected); PM, parental mosaicism; Associated disease phenotypes: ALS, amyotrophic lateral sclerosis; dHMN, distal hereditary motor neuropathy; CMT, Charcot-Marie-Tooth disease; C, cataract; CC, congenital cataract; JC, juvenile cataract; MFM/AxM/DM, myofibrillar/axial/distal myopathy; DCM/RCM/HCM, dilated/restrictive/hypertrophic cardiomyopathy; FP, feet paresthesia; LLS, lower limb spasticity; N/D, no data available. Assumed, though not observed, inheritance is given in parenthesis.

[b] Individuals or families with observed or assumed dominant or semi-dominant disease phenotypes exhibited heterozygosity with both wild-type and mutant alleles being present. The four individuals or families with recessive disease phenotypes were homozygous with regard to the mutant alleles.

[c] Personal communication by Dr. J. Irobi, Antwerp, Belgium.

[d] The mutation p.R140G in HspB1 occurred in both heterozygous and homozygous individuals, suggesting tentatively a semi-dominant inheritance pattern.

[e] The pathogenicity of the HspB3 mutations has been disputed (Adriaenssens et al. 2017; Vendredy et al. 2020).

[f] References:

Adriaenssens E, Geuens T, Baets J, Echaniz-Laguna A, Timmerman V (2017) Novel insights in the disease biology of mutant small heat shock proteins in neuromuscular diseases. Brain 140:2541-2549

Benedetti S, Previtali SC, Coviello S, Scarlato M, Cerri F, Di Pierri E, Piantoni L, Spiga I, Fazio R, Riva N, Natali Sora MG, Dacci P, Malaguti MC, Munerati E, Grimaldi LM, Marrosu MG, De Pellegrin M, Ferrari M, Comi G, Quattrini A, Bolino A (2010). Analyzing histopathological features of rare charcot-marie-tooth neuropathies to unravel their pathogenesis. Arch Neurol 67:1498-1505

Brodehl A, Gaertner-Rommel A, Klauke B, Grewe SA, Schirmer I, Peterschröder A, Faber L, Vorgerd M, Gummert J, Anselmetti D, Schulz U, Paluszkiewicz L, Milting H (2017) The novel αB-crystallin (CRYAB) mutation p.D109G causes restrictive cardiomyopathy. Hum Mutat 38:947-952

Bugiardini E, Rossor AM, Lynch DS, Swash M, Pittman AM, Blake JC, Hanna MG, Houlden H, Holton JL, Reilly MM, Matthews E (2017) Homozygous mutation in *HSPB1* causing distal vacuolar myopathy and motor neuropathy. Neurol Genet 3:e168

Capponi S, Geroldi A, Fossa P, Grandis M, Ciotti P, Gulli R, Schenone A, Mandich P, Bellone E (2011) HSPB1 and HSPB8 in inherited neuropathies: study of an Italian cohort of dHMN and CMT2 patients. J Peripher Nerv Syst 16:287-294

Capponi S, Geuens T, Geroldi A, Origone P, Verdiani S, Cichero E, Adriaenssens E, De Winter V, Bandettini di Poggio M, Barberis M, Chiò A, Fossa P, Mandich P, Bellone E, Timmerman V (2016) Molecular Chaperones in the Pathogenesis of Amyotrophic Lateral Sclerosis: The Role of HSPB1. Hum Mutat 37:1202-1208

Chen J, Liu X, Xu Y, Fan D (2021) Rare variants of HSPB1 are probably associated with amyotrophic lateral sclerosis. Nan Fang Yi Ke Da Xue Xue Bao 41:75-78

Chung KW, Kim SB, Cho SY, Hwang SJ, Park SW, Kang SH, Kim J, Yoo JH, Choi BO (2008) Distal hereditary motor neuropathy in Korean patients with a small heat shock protein 27 mutation. Exp Mol Med 40:304-312

Dierick I, Baets J, Irobi J, Jacobs A, De Vriendt E, Deconinck T, Merlini L, Van den Bergh P, Rasic VM, Robberecht W, Fischer D, Morales RJ, Mitrovic Z, Seeman P, Mazanec R, Kochanski A, Jordanova A, Auer-Grumbach M, Helderman-van den Enden AT, Wokke JH, Nelis E, De Jonghe P, Timmerman V (2008) Relative contribution of mutations in genes for autosomal dominant distal hereditary motor neuropathies: a genotype-phenotype correlation study. Brain 131:1217-1227

Echaniz-Laguna A, Geuens T, Petiot P, Péréon Y, Adriaenssens E, Haidar M, Capponi S, Maisonobe T, Fournier E, Dubourg O, Degos B, Salachas F, Lenglet T, Eymard B, Delmont E, Pouget J, Juntas Morales R, Goizet C, Latour P, Timmerman V, Stojkovic T (2017) Axonal Neuropathies due to Mutations in Small Heat Shock Proteins: Clinical, Genetic, and Functional Insights into Novel Mutations. Hum Mutat 38:556-568

Evgrafov OV, Mersiyanova I, Irobi J, Van Den Bosch L, Dierick I, Leung CL, Schagina O, Verpoorten N, Van Impe K, Fedotov V, Dadali E, Auer-Grumbach M, Windpassinger C, Wagner K, Mitrovic Z, Hilton-Jones D, Talbot K, Martin JJ, Vasserman N, Tverskaya S, Polyakov A, Liem RK, Gettemans J, Robberecht W, De Jonghe P, Timmerman V (2004) Mutant small heat-shock protein 27 causes axonal Charcot-Marie-Tooth disease and distal hereditary motor neuropathy. Nat Genet 36:602-606

Fortunato F, Neri M, Geroldi A, Bellone E, De Grandis D, Ferlini A, Gualandi F (2017) A CMT2 family carrying the P7R mutation in the N- terminal region of the HSPB1 gene. Clin Neurol Neurosurg 163:15-17

Frasquet M, Rojas-García R, Argente-Escrig H, Vázquez-Costa JF, Muelas N, Vílchez JJ, Sivera R, Millet E, Barreiro M, Díaz-Manera J, Turon-Sans J, Cortés-Vicente E, Querol L, Ramírez-Jiménez L, Martínez-Rubio D, Sánchez-Monteagudo A, Espinós C, Sevilla T, Lupo V (2021) Distal hereditary motor neuropathies: Mutation spectrum and genotype-phenotype correlation. Eur J Neurol. 28:1334-1343

Gaeta M, Mileto A, Mazzeo A, Minutoli F, Di Leo R, Settineri N, Donato R, Ascenti G, Blandino A (2012) MRI findings, patterns of disease distribution, and muscle fat fraction calculation in five patients with Charcot-Marie-Tooth type 2F disease. Skeletal Radiol 41:515-524

Ghaoui R, Palmio J, Brewer J, Lek M, Needham M, Evilä A, Hackman P, Jonson PH, Penttilä S, Vihola A, Huovinen S, Lindfors M, Davis RL, Waddell L, Kaur S, Yiannikas C, North K, Clarke N, MacArthur DG, Sue CM, Udd B (2016) Mutations in HSPB8 causing a new phenotype of distal myopathy and motor neuropathy. Neurology 86:391-398

Ho CC, Tai SM, Lee ECN, Mak TSH, Liu TKT, Tang VWL, Poon WT (2017) Rapid identification of pathogenic variants in two cases of Charcot-Marie-tooth disease by gene-panel sequencing. Int J Mol Sci 18:770

Houlden H, Laura M, Wavrant-De Vrièze F, Blake J, Wood N, Reilly MM (2008) Mutations in the HSP27 (HSPB1) gene cause dominant, recessive, and sporadic distal HMN/CMT type 2. Neurology 71:1660-1668

Ikeda Y, Abe A, Ishida C, Takahashi K, Hayasaka K, Yamada M (2009) A clinical phenotype of distal hereditary motor neuronopathy type II with a novel HSPB1 mutation. J Neurol Sci 277: 9-12

Inagaki N, Hayashi T, Arimura T, Koga Y, Takahashi M, Shibata H, Teraoka K, Chikamori T, Yamashina A, Kimura A (2006) Alpha B-crystallin mutation in dilated cardiomyopathy. Biochem Biophys Res Commun 342:379-386

Irobi J, Van Impe K, Seeman P, Jordanova A, Dierick I, Verpoorten N, Michalik A, De Vriendt E, Jacobs A, Van Gerwen V, Vennekens K, Mazanec R, Tournev I, Hilton-Jones D, Talbot K, Kremensky I, Van Den Bosch L, Robberecht W, Van Vandekerckhove J, Van Broeckhoven C, Gettemans J, De Jonghe P, Timmerman V (2004) Hot-spot residue in small heat-shock protein 22 causes distal motor neuropathy. Nat Genet. 36:597-601

James PA, Rankin J, Talbot K (2008) Asymmetrical late onset motor neuropathy associated with a novel mutation in the small heat shock protein HSPB1 (HSP27). J Neurol Neurosurg Psychiatry 79:461-463

Kijima K, Numakura C, Goto T, Takahashi T, Otagiri T, Umetsu K, Hayasaka K (2005) Small heat shock protein 27 mutation in a Japanese patient with distal hereditary motor neuropathy. J Hum Genet 50:473-476

Kolb SJ, Snyder PJ, Poi EJ, Renard EA, Bartlett A, Gu S, Sutton S, Arnold WD, Freimer ML, Lawson VH, Kissel JT, Prior TW (2010) Mutant small heat shock protein B3 causes motor neuropathy: utility of a candidate gene approach. Neurology 74:502-506

Laššuthová P, Šafka Brožková D, Krůtová M, Neupauerová J, Haberlová J, Mazanec R, Dřímal P, Seeman P (2016) Improving diagnosis of inherited peripheral neuropathies through gene panel analysis. Orphanet J Rare Dis 11:118

Lewis-Smith DJ, Duff J, Pyle A, Griffin H, Polvikoski T, Birchall D, Horvath R, Chinnery PF (2016) Novel HSPB1 mutation causes both motor neuronopathy and distal myopathy. Neurol Genet 2(6):e110

Lin KP, Soong BW, Yang CC, Huang LW, Chang MH, Lee IH, AntonellisA, Lee YC (2011) The mutational spectrum in a cohort of Charcot-Marie-Tooth disease type 2 among the Han Chinese in Taiwan. PLoS One 6:e29393

Liu M, Ke T, Wang Z, Yang Q, Chang W, Jiang F, Tang Z, Li H, Ren X, Wang X, Wang T, Li Q, Yang J, Liu J, Wang QK (2006a) Identification of a CRYAB mutation associated with autosomal dominant posterior polar cataract in a Chinese family. Invest Ophthal Vis Sci 47:3461-3466

Liu Y, Zhang X, Luo L, Wu M, Zeng R, Cheng G, Hu B, Liu B, Liang JJ, Shang FA (2006b) A novel alphaB-crystallin mutation associated with autosomal dominant congenital lamellar cataract. Invest Ophthal Vis Sci 47:1069-1075

Luigetti M, Fabrizi GM, Madia F, Ferrarini M, Conte A, Del Grande A, Tasca G, Tonali PA, Sabatelli M (2010) A novel HSPB1 mutation in an Italian patient with CMT2/dHMN phenotype. J Neurol Sci 298:114-117

Maeda K, Idehara R, Hashiguchi A, Takashima H (2014) A family with distal hereditary motor neuropathy and a K141Q mutation of small heat shock protein HSPB1. Intern Med 53:1655-1658

Morelli FF, Verbeek DS, Bertacchini J, Vinet J, Mediani L, Marmiroli S, Cenacchi G, Nasi M, De Biasi S, Brunsting JF, Lammerding J, Pegoraro E, Angelini C, Tupler R, Alberti S, Carra S (2017) Aberrant Compartment Formation by HSPB2 Mislocalizes Lamin A and Compromises Nuclear Integrity and Function. Cell Rep 20:2100-2115

Nakhro K, Park JM, Kim YJ, Yoon BR, Yoo JH, Koo H, Choi BO, Chung KW (2013) A novel Lys141Thr mutation in small heat shock protein 22 (HSPB8) gene in Charcot-Marie-Tooth disease type 2L. Neuromuscul Disord 23:656-663

Nam DE, Nam SH, Lee AJ, Hong YB, Choi BO, Chung KW (2018) Small heat shock protein B3 (HSPB3) mutation in an axonal Charcot-Marie-Tooth disease family. J Peripher Nerv Syst. 23:60-66

Oberstadt M, Mitter D, Classen J, Baum P (2016) Late onset dHMN II caused by c.404C>G mutation in HSPB1 gene. J Peripher Nerv Syst 21:111-113

Pilotto A, Marziliano N, Pasotti M, Grasso M, Costante AM, Arbustini E (2006) AlphaB-crystallin mutation in dilated cardiomyopathies: low prevalence in a consecutive series of 200 unrelated probands. Biochem Biophys Res Commun. 346:1115-1117

Reilich P, Schoser B, Schramm N, Krause S, Schessl J, Kress W, Müller-Höcker J, Walter MC, Lochmuller H (2010) The p.G154S mutation of the alphaB-crystallin gene (CRYAB) causes late-onset distal myopathy. Neuromuscul Disord 20:255-259

Rossor AM, Morrow JM, Polke JM, Murphy SM, Houlden H; INC-RDCRC, Laura M, Manji H, Blake J, Reilly MM (2017) Pilot phenotype and natural history study of hereditary neuropathies caused by mutations in the HSPB1 gene. Neuromuscul Disord 27:50-56

Sacconi S, Feasson L, Antoine JC, Pecheux C, Bernard R, Cobo AM, Casarin A, Salviati L, Desnuelle C, Urtizberea A (2012) A novel CRYAB mutation resulting in multisystemic disease. Neuromusc Disord 22:66-72

Sadeh M, Rahat D, Meiner V, Fellig Y, Arad M, Schueler-Furman O, Hu Y, Li Y, Bönnemann CG, Lossos A (2021) Multi-system neurological disorder associated with a CRYAB variant. Neurogenetics 22:117-125

Safieh LA, Khan AO, Alkuraya FS (2009) Identification of a novel CRYAB mutation associated with autosomal recessive juvenile cataract in a Saudi family. Molec Vis 15:980-984

Scarlato M, Viganò F, Carrera P, Previtali SC, Bolino A (2015) A novel heat shock protein 27 homozygous mutation: widening of the continuum between MND/dHMN/CMT2. J Peripher Nerv Syst 20:419-421

Stancanelli C, Fabrizi GM, Ferrarini M, Cavallaro T, Taioli F, Di Leo R, Russo M, Gentile L, Toscano A, Vita G, Mazzeo A (2015) Charcot-Marie-Tooth 2F: phenotypic presentation of the Arg136Leu HSP27 mutation in a multigenerational family. Neurol Sci 36:1003-1006

Tang B, Liu X, Zhao G, Luo W, Xia K, Pan Q, Cai F, Hu Z, Zhang C, Chen B, Zhang F, Shen L, Zhang R, Jiang H (2005a) Mutation analysis of the small heat shock protein 27 gene in Chinese patients with Charcot-Marie-Tooth disease. Arch Neurol 62:1201-1207

Tang BS, Zhao GH, Luo W, Xia K, Cai F, Pan Q, Zhang RX, Zhang FF, Liu XM, Chen B, Zhang C, Shen L, Jiang H, Long ZG, Dai HP (2005b) Small heat-shock protein 22 mutated in autosomal dominant Charcot-Marie-Tooth disease type 2L.Hum Genet 116:222-224

Vendredy L, Adriaenssens E, Timmerman V (2020) Small heat shock proteins in neurodegenerative diseases. Cell Stress Chaperones 25:679-699

Vicart P, Caron A, Guicheney P, Li Z, Prévost MC, Faure A, Chateau D, Chapon F, Tomé F, Dupret JM, Paulin D, Fardeau M (1998) A missense mutation in the alphaB-crystallin chaperone gene causes a desmin-related myopathy. Nat Genet 20:92-95

Ylikallio E, Johari M, Konovalova S, Moilanen JS, Kiuru-Enari S, Auranen M, Pajunen L, Tyynismaa H (2014) Targeted next-generation sequencing reveals further genetic heterogeneity in axonal Charcot-Marie-Tooth neuropathy and a mutation in HSPB1. Eur J Hum Genet 22:522-527
