# Supplementary material for: Human HspB1, HspB3, HspB5 and HspB8: Shaping these disease factors during vertebrate evolution
Source: Cell Stress Chaperones. 2022 Jun 9;27(4):309–23. doi: 10.1007/s12192-022-01268-y (PMC9346038; doi:10.1007/s12192-022-01268-y)
Supplement: Supplementary file 3 — (PDF 148 KB) [file 12192_2022_1268_MOESM3_ESM.pdf]

Online Supplemental Materials

Table S2. List of sHSP mRNAs (GeneBank and ENSMBLE identifiers) as used for the FEL analysis, the species of origin and their taxonomic ranking

If the coding part of the sHSP mRNAs were embedded in larger sequence blocks, Expasy translation tool (https://web.expasy.org/translate/) was used to identify the coding parts of the sequences.

HspB1
Gnathostomata: Teleostomi: Euteleostomi: Sarcopterygii: Dipnotetrapodomorpha: Tetrapoda: Amniota: Mammalia: Theria: Eutheria: Boreoeutheria: Euarchontoglires: Primates
001. CR536489.1 (Homo sapiens)
002. XM_002743949.4 (Callithrix jacchus)
003. ENSCANT00000043608.1 (Colobus angolensis)
004. XM_017533485.1 (Cebus capucinus)
005. ENSCATT00000043996.1 (Cerocebus atys)
006. XM_004045617.2 (Gorilla gorilla)
007. NM_001283885.1 (Macaca fascicularis)
008. NM_001260949.2 (Macaca mulatta)
009. XM_012759201.2 (Microcebus murinus)
010. XM_012498702.1 (Nomascus leucogenys)
011. XM_003794184.3 (Otolemur garnettii)
012. ENSPPAT00000052328.1 (Pan paniscus)
013. GABE01006326.1 (Pan troglodytes)
014. XM_003895845.2 (Papio anubis)
015. XM_024249722.1 (Pongo abelii)
016. XM_012640279.1 (Propithecus coquereli)
017. XM_010387433.1 (Rhinopithecus roxellana)
018. AY518309.1 (Saguinus oedipus)
Euarchontoglires: Glires
019. XM_020156395.1 (Castor canadensis)
020. XM_003470110.4 (Cavia porcellus)
021. XM_005415130.2 (Chinchilla lanigera)
022. XM_010619058.2 (Fukomys damarensis)
023. XM_004840094.3 (Heterocephalus glaber)
024. XM_004666274.1 (Jaculus jaculus)
025. XM_015490689.1 (Marmota marmota)
026. XM_021649341.1 (Meriones unguiculatus)
027. XM_005080371.3 (Mesocricetus auratus)
028. XM_005344605.3 (Microtus ochrogaster)
029. NM_013560.2 (Mus musculus)
030. XM_021186957.1 (Mus pahari)
031. MGP_SPRETEiJ_T0074122.1 (Mus spretus)
032. XM_008832266.2 (Nannospalax galili)
033. XM_004587193.1 (Ochotona princeps)

034. XM_004630450.2 (Octodon degus)
035. XM_002711905.3 (Oryctolagus cuniculus)
036. XM_006971277.2 (Peromyscus maniculatus)
037. NM_031970.4 (Rattus norvegicus)

Euarchontoglires: Scandentia

038. XM_014590610.1 (Tupaia chinensis)

Euarchontoglires: Dermoptera: Cynocephalidae

039. XM_008594581.1 (Galeopterus variegatus)

Boreoeutheria: Laurasiatheria

040. NM_001304892.1 (Ailuropoda melanoleuca)
041. XM_007186570.1 (Balaenoptera acutorostrata)
042. KX784505.1 (Bos indicus)
043. NM_001025569.1 (Bos taurus)
044. XM_010980735.1 (Camelus dromedarius)
045. NM_001003295.2 (Canis lupus)
046. XM_004442133.2 (Ceratotherium simum)
047. XM_004688019.2 (Condylura cristata)
048. XM_022588028.1 (Delphinapterus leucas)
049. XM_022500951.1 (Enhydra lutris)
050. XM_008141457.1 (Eptesicus fuscus)
051. XM_001504478.4 (Equus caballus)
052. XM_007517945.2 (Erinaceus europaeus)
053. XM_003998540.5 (Felis catus)
054. XM_007468802.1 (Lipotes vexillifer)
055. XM_017654032.1 (Manis javanica)
056. XM_004761627.2 (Mustela putorius)
057. XM_005879150.2 (Myotis brandtii)
058. XM_014455390.2 (Myotis lucifugus)
059. XM_021700598.1 (Neomonachus schauinslandi)
060. XM_004399079.2 (Odobenus rosmarus)
061. XM_004268817.2 (Orcinus orca)
062. XM_012123070.2 (Ovis aries)
063. XM_019421634.1 (Panthera pardus)
064. XM_006918567.3 (Pteropus alecto)
065. XM_007103974.2 (Physeter catodon)
066. XM_011371550.2 (Pteropus vampyrus)
067. XM_019752763.1 (Rhinolophus sinicus)
068. XM_016146541.1 (Rousettus aegyptiacus)
069. XM_004620853.1 (Sorex araneus)
070. NM_001007518.1 (Sus scrofa)
071. XM_026516042.1 (Ursus arctos)

Eutheria: Afrotheria

072. XM_004705622.1 (Echinops telfairi)
073. XM_007940206.1 (Orycteropus afer)
074. ENSPCAT00000013780.1 (Procavia capensis)

075. XM_004391282.2 (Trichechus manatus)
Theria: Metatheria
076. ENSMEUT00000014075.1 (Macropus eugenii)
077. XM_001378935.4 (Monodelphis domestica)
078. ENSSHAT00000014800.1 (Sarcophilus harrisii)
Eutheria: Xenarthra
079. XM_004448274.3 (Dasypus novemcinctus)
Amniota: Sauropsida: Sauria
080. XM_006260278.3 (Alligator mississippiensis)
081. NM_001287309.1 (Alligator mississippiensis)
082. XM_006033234.3 (Alligator sinensis)
083. XM_027471928.1 (Anas platyrhynchos)
084. XM_011581588.1 (Aquila chrysaetos)
085. XM_005284435.3 (Chrysemys picta)
086. XM_005501958.3 (Columba livia)
087. XM_015881044.1 (Coturnix japonica)
088. XM_019545392.1 (Crocodylus porosus)
089. XM_009560237.1 (Cuculus canorus)
090. XM_026107252.1 (Dromaius novaehollandiae)
091. XM_005439800.1 (Falco cherrug)
092. XM_005240472.1 (Falco peregrinus)
093. XM_005056512.2 (Ficedula albicollis)
094. NM_205290.1 (Gallus gallus)
095. XM_015408020.1 (Gekko japonicus)
096. XM_010573540.1 (Haliaeetus leucocephalus)
097. XM_017828875.1 (Lepidothrix coronata)
098. XM_026050202.1 (Nothoprocta perdicaria)
099. XM_015646306.1 (Parus major)
100. XM_025181738.1 (Pelodiscus sinensis)
101. XM_015823845.1 (Protobothrops mucrosquamatus)
102. XM_005525843.2 (Pseudopodoces humilis)
103. XM_009094688.2 (Serinus canaria)
104. XM_002194667.3 (Taeniopygia guttata)
105. XM_024207685.2 (Terrapene mexicana)
Tetrapoda: Amphibia: Batrachia: Anura
106. XM_018571094.1 (Nanorana parkeri)
107. NM_001093816.1 (Xenopus laevis)
108. NM_001079349.1 (Xenopus tropicalis)
Sarcopterygii: Coelacanthimorpha: Coelacanthiformes: Coelacanthidae
109. XM_005992232.2 (Latimeria chalumnae)
Teleostomi: Euteleostomi: Actinopterygii: Actinopteri: Neopterygii
110. XM_022205999.1 (Acanthochromis polyacanthus)
111. XM_023269342.1 (Amphiprion ocellaris)
112. ENSACLT00000008013.1 (Astatotilapia calliptera)
113. XM_007255827.3 (Astyanax mexicanus)

114. XM_014019664.1 (Austrofundulus limnaeus)
115. KT359728.1 (Clarias batrachus)
116. XM_026240617.1 (Carassius auratus)
117. ENSCSET00000017302.1 (Cynoglossus semilaevis)
118. XM_015383644.1 (Cyprinodon variegatus)
119. NM_001008615.2 (Danio rerio)
120. XM_027010259.1 (Electrophorus electricus)
121. ENSGMOT00000013588.1 (Gadus morhua)
122. ENSGAFT00000022102.1 (Gambusia affinis)
123. ENSGACT00000026785.1 (Gasterosteus aculeatus)
124. ENSHCOT00000014698.1 (Hippocampus comes)
125. XM_017460709.1 (Ictalurus punctatus)
126. XM_017412492.2 (Kryptolebias marmoratus)
127. ENSLBET00000008022.1. Labrus bergylta)
128. NM_001303311.1 (Larimichthys crocea)
129. XM_018692336.1 (Lates calcarifer)
130. XM_006640987.2 (Lepisosteus oculatus)
131. ENSMAMT00000009140.1 (Mastacembelus armatus)
132. XM_004557456.4 (Maylandia zebra)
133. ENSMMOT00000007488.1 (Mola mola)
134. ENSMALT00000001108.1 (Monopterus albus)
135. XM_015961780.1 (Nothobranchius furzeri)
136. NM_001279530.1 (Oreochromis niloticus)
137. XM_026921457.1 (Pangasianodon hypophthalmus)
138. XM_020085355.1 (Paralichthys olivaceus)
139. ENSPKIT00000014737.1 (Paramormyrops kingsleyae)
140. XM_014989045.1 (Poecilia mexicana)
141. ENSPLAT00000000657.1 (Poecilia latipinna)
142. ENSPFOT00000008950.1 (Poecilia latipinna)
143. ENSPRET00000012065.1 (Poecilia reticulata)
144. ENSPNYT00000022694.1 (Pundamilia nyererei)
145. ENSPNAT00000009103.1 (Pygocentrus nattereri)
146. ENSSMAT00000000761.1 (Scophthalmus maximus)
147. ENSSLDT00000026576.1 (Seriola lalandi)
148. XM_027132842.1 (Tachysurus fulvidraco)
149. ENSTNIT00000011572.1 (Tetraodon. nigroviridis)
150. ENSXCOT00000007329.1 (Xiphophorus couchianus)
151. XM_005800786.3 (Xiphophorus maculatus)

Gnathostomata: Chondrichthyes

152. XM_007896558.1 (Callorhinchus milii)

==

HspB3

Gnathostomata: Teleostomi: Euteleostomi: Sarcopterygii: Dipnotetrapodomorpha: Tetrapoda: Amniota: Mammalia: Theria: Eutheria: Boreoeutheria: Euarchontoglires: Primates

001. NM_006308.2 (Homo sapiens)
002. XM_008992067.3 (Callithrix jacchus)
003. XM_008057475.2 (Carlito syrichta)
004. XM_017533213.1 (Cebus capucinus)
005. XM_012086998.1 (Cercocebus atys)
006. XM_007972294.1 (Chlorocebus sabaeus)
007. XM_011939355.1 (Colobus angolensis)
008. XM_004058861.2 (Gorilla gorilla)
009. XM_005556891.2 (Macaca fascicularis)
010. XM_001096576 (Macaca mulatta)
011. XM_012756743 (Microcebus murinus)
012. XM_003276527 (Nomascus leucogenys)
013. XM_003782777 (Otolemur garnettii)
014. XM_003827356.2 (Pan paniscus)
015. XM_517764.6 (Pan troglodytes)
016. XM_003899666.3 (Papio anubis)
017. XM_002815553.2 (Pongo abelii)
018. XM_012655284.1 (Propithecus coquereli)
019. XM_010353161.1 (Rhinopithecus roxellana)
020. XM_003925862.1 (Saimiri boliviensis)

Euarchontoglires: Glires

021. XM_020172000.1 (Castor canadensis)
022. XM_003470185.4 (Cavia porcellus)
023. XM_005392716.1 (Chinchilla lanigera)
024. XM_027401441.1 (Cricetulus griseus)
025. XM_013018573.1 (Dipodomys ordii)
026. XM_010626859.2 (Fukomys damarensis)
027. XM_004848525.2 (Heterocephalus glaber)
028. XM_004664944.1 (Jaculus jaculus)
029. XM_015497380.1 (Marmota marmota marmota)
030. XM_021638917.1 (Meriones unguiculatus)
031. XM_005065524.3 (Mesocricetus auratus)
032. XM_005356816.3 (Microtus ochrogaster)
033. 041 NM_019960.2 (Mus musculus)
034. XM_021208803.1 (Mus pahari)
035. XM_008832949.1 (Nannospalax galili)
036. XM_004583794.1 (Ochotona princeps)
037.XM_004623098.2 (Octodon degus)
038. XM_002714030.3 (Oryctolagus cuniculus)
039. XM_016002221.1 (Peromyscus maniculatus bairdii)
040. NM_031750.1 (Rattus norvegicus)

Euarchontoglires: Scandentia
041. XM_006149012.1 (Tupaia chinensis)
Euarchontoglires: Dermoptera: Cynocephalidae
042. XM_008586990.1 (Galeopterus variegatus)
Boreoeutheria: Laurasiatheria
043. XM_002927469.3 (Ailuropoda melanoleuca)
044. XM_007195583.1 (Balaenoptera acutorostrata)
045. NM_001046570.2 (Bos taurus)
046. XM_006185923.2 (Camelus ferus)
047. XM_010977776.1 (Camelus dromedarius)
048. XM_005619327.3 (Canis lupus)
049. XM_014781626.1 (Ceratotherium simum)
050. XM_012721798.1 (Condylura cristata)
051. XM_022551605.1 (Delphinapterus leucas)
052. XM_022493473.1 (Enhydra lutris)
053. XM_005604272.3 (Equus caballus)
054. XM_007518898.2 (Erinaceus europaeus)
055. XM_006928019.4 (Felis catus)
056. XM_019641426.1 (Hipposideros armiger)
057. XM_007469286.1 (Lipotes vexillifer)
058. XM_017649540.1 (Manis javanica)
059. XM_016199828.1 (Miniopterus natalensis)
060. XM_004737891.2 (Mustela putorius)
061. XM_004413285.2 (Odobenus rosmarus)
062. XM_004275133.1 (Orcinus orca)
063. XM_012135022.2 (Ovis aries musimon)
064. XM_019464339.1 (Panthera pardus)
065. XM_007076644.2 (Panthera tigris)
066. XM_006912453.1 (Pteropus aletco)
067. XM_028492905.1 (Physeter catodon)
068. XM_028492906.1 (Physeter catodon)
069. XM_011380607.1 (Pteropus vampyrus)
070. XM_016142353.1 (Rousettus aegyptiacus)
071. XM_004608527.1 (Sorex araneus)
072. XM_003133967.4 (Sus scrofa)
073. XM_026510651.1 (Ursus arctos)
074. XM_008692539.1 (Ursus maritimus)
075. XM_015240724.1 (Vicugna pacos)
Eutheria: Afrotheria
076. XM_004703719.1 (Echinops telfairi)
077. XM_006889568.1 (Elephantulus edwardii)
078. XM_003408031.2 (Loxodonta africana)
079. XM_007951536.1 (Orycteropus afer)
080. ENSPCAT00000013456.1 (Procavia capensis)
081. XM_004380462.1 (Trichechus manatus latirostis)

Theria: Metatheria
082. ENSMEUT00000015135.1 (Macropus eugenii)
083. XM_001380851.2 (Monodelphis domestica)
084. XM_003763125.1 (Sarcophilus harrisii)
Eutheria: Xenarthra
085. XM_004483480.3 (Dasypus novemcinctus)
Amniota: Sauropsida: Sauria
086. XM_006017287.2 (Alligator sinensis)
087. XM_003216187.3 (Anolis carolinensis)
088. XM_011596908.1 (Aquila chrysaetos canadensis)
089. XM_014964827.1 (Calidirs pugnax)
090. XM_008500142.1 (Calypte anna)
091. XM_005282165.2 (Chrysemys picta bellii)
092. XM_021285444.1 (Columba livia)
093. XM_017744316.1 (Corvus brachyrhynchos)
094. XM_015848720.1 (Coturnix japonica)
095. XM_019532496 (Crocodylus porosus)
096. XM_009555858.1 (Cuculus canorus)
097. XM_026120936 (Dromaius novaehollandiae)
098. 131. XM_005443917.2 (Falco cherrug)
099. XM_005241994 (Falco peregrinus)
100. XM_005060621 (Ficedula albicollis)
101. XM_001231557 (Gallus gallus)
102. XM_015419159 (Gekko japonicus)
103. XM_017811136 (Lepidothrix coronata)
104. XM_021553896 (Lonchura striata domestica)
105. XM_009461137 (Nipponia nippon)
106. XM_015615583 (Parus major)
107. XM_006139541.3 (Pelodiscus sinensis)
108. XM_015822883 (Protobothrops mucrosquamatus)
109. XM_014259875 (Pseudopodoces humilis)
110. XM_018920640 (Serinus canaria)
111. XM_002195233.1 (Taeniopygia guttata)
112. XP_024065889.2 (Terrapene carolina triunguis)
113. XM_026795640 (Zonotrichia albicollis)
Tetrapoda: Amphibia: Batrachia: Anura
114. XM_018563319 (Nanorana parkeri)
115. XM_018226893 (Xenopus laevis)
116. XM_002941028.4 (Xenopus tropicalis)
Sarcopterygii: Coelacanthimorpha: Coelacanthiformes: Coelacanthidae
117. XM_006006980.2 (Latimeria chalumnae)
Teleostomi: Euteleostomi: Actinopterygii: Actinopteri: Neopterygii
118. XM_007250482.2 (Astyanax mexicanus)
119. XM_019095081.1 (Cyprinus carpio)
120. NM_001099452.1 (Danio rerio)

121. XM_020052862.1 (Esox lucius)
122. XM_015365087.1 (Lepisosteus oculatus)
123. XM_023820693.1 (Paramormyrops kingsleyae)
124. XM_017705104.1 (Pygocentrus nattereri)
125. XM_016561090.1 (Sinocyclocheilus anshuiensis)
126. XM_016290694.1 (Sinocyclocheilus grahami)
127. XM_016561090.1 (Sinocyclocheilus rhinocerous)

Actinopteri: Chondrostei: Acipenseriformes: Acipenseroidei: Acipenseridae: Acipenserinae: Acipenserini

128. MH777919.1 (Acipenser oxyrinchus)

Gnathostomata: Chondrichthyes

129. XM_007892194.1 (Callorhinchus milii)
130. XM_020511366.1 (Rhincodon typus)

==

HspB5

Gnathostomata: Teleostomi: Euteleostomi: Sarcopterygii: Dipnotetrapodomorpha: Tetrapoda: Amniota: Mammalia: Theria: Eutheria: Boreoeutheria: Euarchontoglires: Primates

001. NM_001885.2 (Homo sapiens)
002. XM_011926733.1 (Colobus angolensis)
003. XM_002754383.3 (Callithrix jacchus)
004. XM_008065587.2 (Carlito syrichta)
005. XM_017517682.1 (Cebus capucinus)
006. XM_012066401.1 (Cercocebus atys)
007. XM_008020848.1 (Chlorocebus sabaeus)
008. XM_004052118.2 (Gorilla gorilla)
009. XM_015435350.1 (Macaca fascicularis)
010. NM_001260901.1 (Macaca mulatta)
011. XM_012749592.2 (Microcebus murinus)
012. XM_004090563.2 (Nomascus leucogenys)
013. XM_003794732.3 (Otolemur garnettii)
014. XM_003805460.4 (Pan paniscus)
015. XM_016921980.2 (Pan troglodytes)
016. XM_009247045.2 (Pongo abelii)
017 XM_012659198.1 (Propithecus coquereli)
018. XM_010382343.1 (Rhinopithecus roxellana)
019. XM_010334574.1 (Saimiri boliviensis)

Euarchontoglires: Glires

020. XM_020175744.1 (Castor canadensis)
021. NM_001173076.1 (Cavia porcellus)
022. XM_005378100.2 (Chinchilla lanigera)
023. XM_016968238.1 (Cricetulus griseus)
024. XM_010610684.2 (Fukomys damarensis)
025. NM_001279857.2 (Heterocephalus glaber)
026. XM_004666484.2 (Jaculus jaculus)
027. XM_015489896.1 (Marmota marmota marmota)
028. XM_021658661.1 (Meriones unguiculatus)
029. XM_013113005.2 (Mesocricetus auratus)
030. XM_005347316.3 (Microtus ochrogaster)

031. NM_001289785.1 (Mus musculus)
032. XM_021206500.1 (Mus pahari)
033. XM_008832795.1 (Nannospalax galili)
034. XM_012926934.1 (Ochotona princeps)
035. XM_004646354.3 (Octodon degus)
036. NM_001082407.1 (Oryctolagus cuniculus)
037. XM_015987087.1 (Peromyscus maniculatus)
038. NM_012935.4 (Rattus norvegicus)
Euarchontoglires: Scandentia
039. XM_006150984.2 (Tupaia chinensis)
Euarchontoglires: Dermoptera: Cynocephalidae
040. XM_008577609.1 (Galeopterus variegatus)
Boreoeutheria: Laurasiatheria
041. NM_001304874.1 (Ailuropoda melanoleuca)
042. XM_019975270.1 (Bos indicus)
043. NM_174290.2 (Bos taurus)
044. XM_006190373.2 (Camelus ferus)
045. KJ174932.1 (Camelus dromedarius)
046. XM_857165.5 (Canis lupus)
047. XM_004427339.2 (Ceratotherium simum)
048. XM_004689273.2 (Condylura cristata)
049. XM_022561589.1 (Delphinapterus leucas)
050. XM_022512857.1 (Enhydra lutris)
051. XM_008150161.1 (Eptesicus fuscus)
052. XM_001501779.5 (Equus caballus)
053. XM_007537847.2 (Erinaceus europaeus)
054. XM_011286345.3 (Felis catus)
055. XM_019653686.1 (Hipposideros armiger)
056. XM_007464652.1 (Lipotes vexillifer)
057. XM_017659392.1 (Manis javanica)
058. XM_004749799.2 (Mustela putorius)
059. XM_012567097.1 (Odobenus rosmarus)
060. XM_004273414.2 (Orcinus orca)
061. NM_001012457.2 (Ovis aries)
062. XM_019469186.1 (Panthera pardus)
063. XM_015542578.1 (Panthera tigris)
064. XM_007128267.2 (Physeter catodon)
065. XM_006912821.1 (Pteropus alecto)
066. XM_011365409.2 (Pteropus vampyrus)
067. XM_016149055.1 (Rousettus aegyptiacus)
068. XM_004604685.2 (Sorex araneus)
069. XM_021062779.1 (Sus scrofa)
070. XM_008690086.1 (Ursus maritimus)
071. XM_006217871.2 (Vicugna pacos)
Eutheria: Afrotheria
072. XM_006833817.1 (Chrysochloris asiatica)
073. XM_004708973.2 (Echinops telfairi)
074. XM_006890772.1 (Elephantulus edwardii)
075. XM_003415624.3 (Loxodonta africana)

076. XM_007936553.1 (Orycteropus afer)
077. ENSPCAT00000001647.1 (Procavia capensis)
078. XM_004382499.3 (Trichechus manatus)

Theria: Metatheria

079. XM_001371207.3 (Monodelphis domestica)
080. XM_020989782.1 (Phascolarctos cinereus)
081. XM_003764224.2 (Sarcophilus harrisii)

Eutheria: Xenarthra

082. XM_004481144.3 (Dasypus novemcinctus)

Amniota: Sauropsida: Sauria

083. XM_006271349.3 (Alligator mississippiensis)
084. NM_001310366.1 (Anas platyrhynchos)
085. XM_008121072.2 (Anolis carolinensis)
086. XM_010169584.1 (Antrostomus carolinensis)
087. XM_009870055.1 (Apaloderma vittatum)
088. XM_011601882.1 (Aquila chrysaetos)
089. XM_026863824.1 (Athene cunicularia)
090. XM_010298661.1 (Balearica regulorum)
091. XM_010142606.1 (Buceros rhinoceros)
092. XM_014949523.1 (Calidris pugnax)
093. XM_008492420.1 (Calypte anna)
094. XM_009696584.1 (Cariama cristata)
095. XM_009997410.1 (Chaetura pelagica)
096. XM_009882443.1 (Charadrius vociferus)
097. XM_005291386.3 (Chrysemys picta)
098. XM_021285292.1 (Columba livia)
099. XM_008636923.2 (Corvus brachyrhynchos)
100. XM_015883834.1 (Coturnix japonica)
101. XM_019547110.1 (Crocodylus porosus)
102. XM_009569900.1 (Cuculus canorus)
103. XM_026105876.1 (Dromaius novaehollandiae)
104. XM_009638415.1 (Egretta garzetta)
105. XM_005440198.1 (Falco cherrug)
106. XM_005236602.1 (Falco peregrinus)
107. XM_005058800.1 (Ficedula albicollis)
108. NM_205176.1 (Gallus gallus)
109. XM_009810695.1 (Gavia stellata)
110. XM_015415588.1 (Gekko japonicus)
111. XM_010575003.1 (Haliaeetus leucocephalus)
112. XM_009917256.1 (Haliaeetus albicilla)
113. XM_017809764.1 (Lepidothrix coronata)
114. XM_021530106.1 (Lonchura striata)
115. XM_008935459.1 (Merops nubicus)
116. XM_009472271.1 (Nipponia nippon)
117. XM_026041046.1 (Nothoprocta perdicaria)
118. XM_009944637.1 (Opisthocomus hoazin)
119. XM_015649982.1 (Parus major)
120. XM_009485675.1 (Pelecanus crispus)
121. XM_005531058.2 (Pseudopodoces humilis)

122. XM_009098028.2 (Serinus canaria)
123. XM_002192884.3 (Taeniopygia guttata)
124. XM_024207189.2 (Terrapene mexicana)
Tetrapoda: Amphibia: Batrachia: Anura
125. XM_018564029.1 (Nanorana parkeri)
126. XM_018225915.1 (Xenopus laevis)
127. XM_002932918.4 (Xenopus tropicalis)
Sarcopterygii: Coelacanthimorpha: Coelacanthiformes: Coelacanthidae
128. XM_005987256.2 (Latimeria chalumnae)
Teleostomi: Euteleostomi: Actinopterygii: Actinopteri: Neopterygii
129. ENSAPOT00000005575.1 (Acanthochromis polyacanthus)
130. ENSAMXT00000007540.2 (Astyanax mexicanus)
131. XM_026212446.1 (Carassius auratus)
132. XM_012821983.1 (Clupea harengus)
133. XM_019095482.1 (Cyprinus carpio)
134. NM_131157.1 (Danio rerio)
135. XM_027031825.1 (Electrophorus electricus)
136. ENSIPUT00000034725.1 (Ictalurus punctatus)
137. XM_006642147.2 (Lepisosteus oculatus)
138. XM_026921851.1 (Pangasianodon hypophthalmus)
139. XM_023813558.1 (Paramormyrops kingsleyae)
140. XM_017699999.1 (Pygocentrus nattereri)
141. XM_018755058.1 (Scleropages formosus)
142. XM_016490540.1 (Sinocyclocheilus anshuiensis)
143. XM_016295944.1 (Sinocyclocheilus grahami)
144. XM_016518971.1 (Sinocyclocheilus rhinocerous)
145. XM_027178619.1 (Tachysurus fulvidraco)
Gnathostomata: Chondrichthyes
146. XM_007911619.1 (Callorhinchus milii)
147. XM_020530352.1 (Rhincodon typus)
==

HspB8

Gnathostomata: Teleostomi: Euteleostomi: Sarcopterygii: Dipnotetrapodomorpha: Tetrapoda: Amniota: Mammalia: Theria: Eutheria: Boreoeutheria: Euarchontoglires: Primates
001. NM_014365.2 (Homo sapiens)
002. ENSCJAT00000017713 (Callithrix jacchus)
003. XM_008053630.1 (Carlito syrichta)
004. XM_017529563.1 (Cebus capucinus)
005. ENSCSAT00000000316 (Chlorocebus sabaeus)
006. ENSGGOT00000013431 (Gorilla gorilla)
007. XM_005572374.2 (Macaca fascicularis)
008. ENSMMUT00000029873 (Macaca mulatta)
009. ENSMICT00000012392 (Microcebus murinus)
010. ENSNLET00000000278 (Nomascus leucogenys)
011. XM_003789928.1 (Otolemur garnettii)
012. ENSPTRT00000010131 (Pan troglodytes)
013. ENSPPYT00000005936 (Pongo abelii)
014. XM_012650230.1 (Propithecus coquereli)
015. XM_003932197.2 (Saimiri boliviensis)

Euarchontoglires: Glires
016. XM_020163619.1 (Castor canadensis)
017. XM_003478311.3 (Cavia porcellus)
018. XM_005396529.2 (Chinchilla lanigera)
019. XM_007630429.2 (Cricetulus griseus)
020. XM_013014338.1 (Dipodomys ordii)
021. XM_010604080.2 (Fukomys damarensis)
022. XM_004843691.2 (Heterocephalus glaber)
023. XM_004664142.1 (Jaculus jaculus)
024. XM_015505174.1 (Marmota marmota)
025. XM_021631814.1 (Meriones unguiculatus)
026. XM_005078985.3 (Mesocricetus auratus)
027. XM_005344327.1 (Microtus ochrogaster)
028. NM_030704.3 (Mus musculus)
029. XM_021186952.1 (Mus pahari)
030. XM_008824419.2 (Nannospalax galili)
031. ENSOPRT00000016991 (Ochotona princeps)
032. XM_004636239.2 (Octodon degus)
033. XM_006970817.2 (Peromyscus maniculatus)
034. NM_053612.2 (Rattus norvegicus)
Euarchontoglires: Scandentia
035. XM_006151554.2 (Tupaia chinensis)
Euarchontoglires: Dermoptera: Cynocephalidae
036. XM_008592760.1 (Galeopterus variegatus)
Boreoeutheria: Laurasiatheria
037. XM_002919673.3 (Ailuropoda melanoleuca)
038. XM_007189481.1 (Balaenoptera acutorostrata)
039. NM_001014955.1 (Bos taurus)
040. XM_006192699.2 (Camelus ferus)
041. NM_001003029.1 (Canis familiaris)
042. XM_004429927.2 (Ceratotherium simum)
043. XM_004690995.2 (Condylura cristata)
044. XM_022597851.1 (Delphinapterus leucas)
045. XM_022517444.1 (Enhydra lutris)
046. XM_008142767.1 (Eptesicus fuscus)
047. XM_001490413.4 (Equus caballus)
048. XM_007528642.2 (Erinaceus europaeus)
049. XM_003994712.5 (Felis catus)
050. XM_019649044.1 (Hipposideros armiger)
051. XM_007464865.1 (Lipotes vexillifer)
052. XM_017666754.1 (Manis javanica)
053. XM_016201564.1 (Miniopterus natalensis)
054. ENSMPUT00000002968 (Mustela putorius)
055. XM_005863777.2 (Myotis brandtii)
056. ENSMLUT00000016852 (Myotis lucifugus)
057. XM_021703393.1 (Neomonachus schauinslandi)
058. XM_004396682.2 (Odobenus rosmarus)
059. XM_004276749.1 (Orcinus orca)

060. XM_012110114.2 (Ovis aries)
061. XM_007079634.2 (Panthera tigris)
062. XM_006908601.2 (Pteropus alecto)
063. XM_007114647.2 (Physeter catodon)
064. ENSPVAT00000017488 (Pteropus vampyrus)
065. XM_016149531.1 (Rousettus aegyptiacus)
066. XM_019753966.1 (Rhinolophus sinicus)
067. XM_004611071.1 (Sorex araneus)
068. XM_001929585.5 (Sus scrofa)
069. XM_008707686.1 (Ursus maritimus)
070. XM_006204799.2 (Vicugna pacos)

Eutheria: Afrotheria

071. XM_006865455.1 (Chrysochloris asiatica)
072. XM_006890415.1 (Elephantulus edwardii)
073. XM_003419267.3 (Loxodonta africana)
074. XM_007938210.1 (Orycteropus afer)
075. XM_004379029.1 (Trichechus manatus)

Theria: Metatheria

076. ENSSHAT00000020774 (Sarcophilus harrisii)

Amniota: Sauropsida: Sauria

077. XM_014593637.2 (Alligator mississippiensis)
078. XM_006027370.2 (Alligator sinensis)
079. ENSACAT00000004096 (Anolis carolinensis)
080. XM_011592666.1 (Aquila chrysaetos)
081. XM_014965486.1 (Calidris pugnax)
082. XM_008497491.1 (Calypte anna)
083. XM_005303256.3 (Chrysemys picta)
084. XM_017746547.1 (Corvus brachyrhynchos)
085. XM_019539248 (Crocodylus porosus)
086. XM_009566591.1 (Cuculus canorus)
087. XM_005445283.1 (Falco cherrug)
088. XM_005233215.1 (Falco peregrinus)
089. XM_005054867.2 (Ficedula albicollis)
090. ENSGALT00000011938 (Gallus gallus)
091. XM_015425324.1 (Gekko japonicus)
092. XM_010574425.1 (Haliaeetus leucocephalus)
093. XM_017814476.1 (Lepidothrix coronata)
094. XM_021549813.1 (Lonchura striata)
095. XM_009463728.1 (Nipponia nippon)
096. XM_006134168.2 (Pelodiscus sinensis)
097. XM_015817475.1 (Protobothrops mucrosquamatus)
098. XM_005524414.2 (Pseudopodoces humilis)
099. XM_009092308.2 (Serinus canaria)
100. XM_002199452.1 (Taeniopygia guttata)
101. XM_005493452.2 (Zonotrichia albicollis)

Tetrapoda: Amphibia: Batrachia: Anura

102. XM_018570531.1 (Nanorana parkeri)
103. NM_001086313.1 (Xenopus laevis)

104. ETT00000054022 (Xenopus silurana)
Sarcopterygii: Coelacanthimorpha: Coelacanthiformes: Coelacanthidae
105. ENSLACT00000015233 (Latimeria chalumnae)
Teleostomi: Euteleostomi: Actinopterygii: Actinopteri: Neopterygii
106. XM_023277314.1 (Amphiprion ocellaris)
107. XM_026344341.1 (Anabas testudineus)
108. XM_014011157.1 (Austrofundulus limnaeus)
109. XM_012820350.1 (Clupea harengus)
110. XM_015403675.1 (Cyprinodon variegatus)
111. XM_019104600.1 (Cyprinus carpio)
112. NM_001100957.2 (Danio rerio)
113. XM_013136582.2 (Esox lucius)
114. ENSGACT00000018865.1 (Gasterosteus aculeatus)
115. XM_017427754.2 (Kryptolebias marmoratus)
116. XM_010732969.2 (Larimichthys crocea)
117. XM_018703469.1 (Lates calcarifer)
118. ENSLOCT00000003798.1 (Lepisosteus oculatus)
119. XM_004566676.5 (Maylandia zebra)
120. XM_006803878.1 (Neolamprologus brichardi)
121. XM_015960520.1 (Nothobranchius furzeri)
122. XM_021606608.1 (Oncorhynchus mykiss)
123. XM_021622152.1 (Oncorhynchus mykiss)
124. XM_020490486.1 (Oncorhynchus kisutch)
125. XM_020475741.1 (Oncorhynchus kisutch)
126. XM_024402897.1 (Oncorhynchus tshawytscha)
127. XM_024418238.1 (Oncorhynchus tshawytscha)
128. XM_003454160 (Oreochromis niloticus)
129. XM_004072074.4 (Oryzias latipes)
130. XM_020099324.1 (Paralichthys olivaceus)
131. ENSPFOT00000014634.1 (Poecilia formosa)
132. XM_014999665.1 (Poecilia mexicana)
133. XM_015059347 (Poecilia latipinna)
134. XM_014170575.1 (Salmo salar)
135. XM_014160033.1 (Salmo salar)
136. XM_024141472.1 (Salvelinus alpinus)
137. XM_024001519.2 (Salvelinus alpinus)
138. XM_022208447.1 (Scanthochromis polyacanthus)
139. XM_016444754.1 (Sinocyclocheilus anshuiensis)
140. XM_016528695.1 (Sinocyclocheilus rhinocerous)
141. XM_008279881.1 (Stegastes partitus)
142. XM_005803859.2 (Xiphophorus maculatus)
Gnathostomata: Chondrichthyes
143. XM_007899064.1 (Callorhinchus milii)
144. XM_020525166.1 (Rhincodon typus)
