# Supplementary material for: Human HspB1, HspB3, HspB5 and HspB8: Shaping these disease factors during vertebrate evolution
Source: Cell Stress Chaperones. 2022 Jun 9;27(4):309–23. doi: 10.1007/s12192-022-01268-y (PMC9346038; doi:10.1007/s12192-022-01268-y)
Supplement: Supplementary file 4 — (PDF 547 KB) [file 12192_2022_1268_MOESM4_ESM.pdf]

## Online Supplemental Materials

## Table S3. FEL analysis of the coding sequences of human HspB1, HspB3, HspB5 and HspB8

abbreviations and symbols: LB, lower bound; MLE, maximum likelihood estimate; UB, upper bound;
ND, not defined; PurS, purifying selection; NE, neutrally evolving (highlighted in gray);
DivS, diversifying positive selection (highlighted in blue); †, codon excluded from further processing

### HspB1

| site | amino acid | alpha | beta | p-value | dN/dS LB | dN/dS MLE (omega) | dN/dS UB | selection |
|---|---|---|---|---|---|---|---|---|
| 1 | M | 0.000 | 0.000 | n/a | n/a | n/a | n/a | ND † |
| 2 | T | 0.939 | 0.180 | 8.21372379E-07 | 0.109 | 0.192 | 0.311 | PurS |
| 3 | E | 0.540 | 0.000 | 7.968937E-07 | 0.000 | 0.000 | 0.032 | PurS |
| 4 | R | 0.362 | 0.010 | 1.18562837E-06 | 0.002 | 0.027 | 0.121 | PurS |
| 5 | R | 0.721 | 0.194 | 0.000365223006 | 0.154 | 0.270 | 0.436 | PurS |
| 6 | V | 0.624 | 0.029 | 3.35163441E-09 | 0.011 | 0.046 | 0.120 | PurS |
| 7 | P | 0.797 | 0.012 | 5.54156721E-12 | 0.001 | 0.015 | 0.067 | PurS |
| 8 | F | 0.000 | 0.042 | n/a | n/a | n/a | n/a | ND † |
| 9 | S | 0.503 | 0.121 | 0.000937649478 | 0.124 | 0.241 | 0.419 | PurS |
| 10 | L | 0.683 | 0.108 | 9.55517424E-05 | 0.082 | 0.158 | 0.275 | PurS |
| 11 | L | 0.731 | 0.103 | 3.71338788E-06 | 0.064 | 0.141 | 0.265 | PurS |
| 12 | R | 0.597 | 0.058 | 7.15216647E-07 | 0.035 | 0.096 | 0.210 | PurS |
| 13 | G | 0.873 | 0.217 | 0.001088209672 | 0.157 | 0.249 | 0.375 | PurS |
| 14 | P | 1.124 | 0.089 | 2.41318965E-10 | 0.034 | 0.079 | 0.155 | PurS |
| 15 | S | 0.293 | 0.048 | 0.03007813909 | 0.065 | 0.164 | 0.336 | PurS |
| 16 | W | 0.000 | 0.011 | n/a | n/a | n/a | n/a | ND † |
| 17 | D | 1.658 | 0.082 | 8.47840054E-09 | 0.024 | 0.050 | 0.090 | PurS |
| 18 | P | 0.893 | 0.000 | 1.11022302E-16 | 0.000 | 0.000 | 0.026 | PurS |
| 19 | F | 0.922 | 0.034 | 6.19930412E-06 | 0.011 | 0.037 | 0.086 | PurS |
| 20 | R | 0.821 | 0.070 | 1.60838709E-09 | 0.033 | 0.085 | 0.174 | PurS |
| 21 | D | 0.422 | 0.035 | 0.006345816688 | 0.026 | 0.083 | 0.197 | PurS |
| 22 | W | 1.640 | 0.044 | 0.063043779148 | 0.008 | 0.027 | 0.063 | PurS |
| 23 | Y | 1.175 | 0.210 | 0.002229217411 | 0.110 | 0.179 | 0.273 | PurS |
| 24 | P | 1.429 | 0.197 | 7.98804699E-08 | 0.080 | 0.138 | 0.220 | PurS |
| 25 | H | 1.311 | 0.446 | 0.002574869012 | 0.232 | 0.340 | 0.482 | PurS |
| 26 | S | 0.983 | 0.071 | 2.32834178E-05 | 0.034 | 0.072 | 0.131 | PurS |
| 27 | R | 1.157 | 0.022 | 3.230749E-14 | 0.003 | 0.019 | 0.059 | PurS |
| 28 | L | 0.431 | 0.084 | 0.001812495929 | 0.088 | 0.195 | 0.367 | PurS |
| 29 | F | 1.224 | 0.025 | 7.8825835E-13 | 0.005 | 0.020 | 0.053 | PurS |
| 30 | D | 1.003 | 0.063 | 5.35074146E-06 | 0.027 | 0.063 | 0.123 | PurS |

| | | | | | | | | |
|---|---|---|---|---|---|---|---|---|
| 31 | Q | 0.197 | 0.042 | 0.11711214556 | 0.066 | 0.212 | 0.497 | NE |
| 32 | A | 0.837 | 0.298 | 0.002596075079 | 0.227 | 0.356 | 0.533 | PurS |
| 33 | F | 1.631 | 0.000 | 0 | 0.000 | 0.000 | 0.010 | PurS |
| 34 | G | 0.731 | 0.021 | 4.22401225E-10 | 0.005 | 0.029 | 0.090 | PurS |
| 35 | L | 0.742 | 0.121 | 0.001216283867 | 0.091 | 0.163 | 0.267 | PurS |
| 36 | P | 1.890 | 0.049 | 0 | 0.008 | 0.026 | 0.061 | PurS |
| 37 | R | 0.830 | 0.173 | 3.48261917E-05 | 0.115 | 0.209 | 0.347 | PurS |
| 38 | L | 0.616 | 0.244 | 0.015391179189 | 0.243 | 0.396 | 0.608 | PurS |
| 39 | P | 1.884 | 0.211 | 6.12143669E-12 | 0.065 | 0.112 | 0.180 | PurS |
| 40 | E | 1.443 | 0.037 | 6.37925934E-10 | 0.008 | 0.026 | 0.061 | PurS |
| 41 | E | 1.798 | 0.074 | 7.62444996E-10 | 0.019 | 0.041 | 0.078 | PurS |
| 42 | W | 1.094 | 0.119 | 0.000937059695 | 0.056 | 0.109 | 0.188 | PurS |
| 43 | S | 1.418 | 0.463 | 0.001172558699 | 0.220 | 0.327 | 0.471 | PurS |
| 44 | Q | 0.912 | 0.283 | 0.00158524613 | 0.199 | 0.311 | 0.463 | PurS |
| 45 | W | 18.963 | 0.051 | 9.46250545E-09 | 0.001 | 0.003 | 0.006 | PurS |
| 46 | L | 0.981 | 0.132 | 8.88928682E-07 | 0.069 | 0.134 | 0.233 | PurS |
| 47 | G | 1.097 | 0.164 | 4.01311001E-05 | 0.089 | 0.149 | 0.233 | PurS |
| 48 | G | 0.681 | 0.352 | 0.088595452513 | 0.347 | 0.517 | 0.742 | PurS |
| 49 | S | 0.781 | 0.132 | 0.009818974422 | 0.076 | 0.170 | 0.327 | PurS |
| 50 | S | 1.565 | 0.234 | 2.75315831E-06 | 0.093 | 0.150 | 0.227 | PurS |
| 51 | W | 24.794 | 0.022 | 0.007826437672 | 0.000 | 0.001 | 0.003 | PurS |
| 52 | P | 1.470 | 0.012 | 0 | 0.000 | 0.008 | 0.037 | PurS |
| 53 | G | 1.174 | 0.022 | 3.44169138E-15 | 0.003 | 0.019 | 0.058 | PurS |
| 54 | Y | 1.447 | 0.010 | 5.2613469E-13 | 0.000 | 0.007 | 0.030 | PurS |
| 55 | V | 0.692 | 0.259 | 0.02387153567 | 0.235 | 0.373 | 0.563 | PurS |
| 56 | R | 1.859 | 0.014 | 0 | 0.000 | 0.008 | 0.032 | PurS |
| 57 | P | 1.291 | 0.141 | 3.74772546E-10 | 0.056 | 0.109 | 0.189 | PurS |
| 58 | L | 0.884 | 0.394 | 0.019165037724 | 0.297 | 0.446 | 0.643 | PurS |
| 59 | P | 0.818 | 0.393 | 0.038630826722 | 0.306 | 0.480 | 0.720 | PurS |
| 60 | P | 3.144 | 0.440 | 8.17027557E-11 | 0.091 | 0.140 | 0.207 | PurS |
| 61 | A | 2.526 | 0.590 | 0.000108714066 | 0.152 | 0.234 | 0.350 | PurS |
| 62 | A | 1.625 | 0.675 | 0.01265092141 | 0.287 | 0.416 | 0.586 | PurS |
| 63 | I | 1.372 | 0.524 | 0.022100438814 | 0.262 | 0.382 | 0.540 | PurS |
| 64 | E | 2.359 | 0.680 | 0.004997066307 | 0.211 | 0.288 | 0.388 | PurS |
| 65 | S | 2.277 | 0.898 | 0.002910243202 | 0.294 | 0.394 | 0.527 | PurS |
| 66 | P | 1.381 | 0.250 | 4.34550874E-07 | 0.108 | 0.181 | 0.284 | PurS |
| 67 | A | 3.759 | 0.581 | 4.95629754E-07 | 0.107 | 0.155 | 0.218 | PurS |
| 68 | V | 1.431 | 0.654 | 0.022452356004 | 0.322 | 0.457 | 0.632 | PurS |
| 69 | A | 1.612 | 0.405 | 7.90076351E-06 | 0.165 | 0.251 | 0.367 | PurS |
| 70 | A | 1.700 | 0.690 | 0.001617284056 | 0.287 | 0.406 | 0.562 | PurS |

| | | | | | | | | |
|---|---|---|---|---|---|---|---|---|
| 71 | P | 1.423 | 0.290 | 2.78809933E-06 | 0.127 | 0.204 | 0.310 | PurS |
| 72 | A | 0.991 | 0.567 | 0.139144063594 | 0.401 | 0.572 | 0.792 | NE |
| 73 | Y | 0.896 | 0.229 | 0.004462513597 | 0.162 | 0.256 | 0.384 | PurS |
| 74 | S | 1.260 | 0.206 | 5.98161281E-07 | 0.100 | 0.164 | 0.253 | PurS |
| 75 | R | 1.448 | 0.120 | 1.29263267E-12 | 0.041 | 0.083 | 0.148 | PurS |
| 76 | A | 1.106 | 0.111 | 5.51544521E-09 | 0.048 | 0.101 | 0.184 | PurS |
| 77 | L | 0.529 | 0.075 | 4.86602329E-05 | 0.056 | 0.142 | 0.290 | PurS |
| 78 | S | 0.824 | 0.159 | 0.000308674892 | 0.112 | 0.193 | 0.308 | PurS |
| 79 | R | 1.214 | 0.035 | 5.88418203E-15 | 0.007 | 0.029 | 0.075 | PurS |
| 80 | Q | 2.338 | 0.021 | 3.941292E-14 | 0.001 | 0.009 | 0.027 | PurS |
| 81 | L | 0.958 | 0.085 | 6.21229813E-08 | 0.040 | 0.089 | 0.167 | PurS |
| 82 | S | 0.340 | 0.032 | 0.008181549751 | 0.029 | 0.093 | 0.216 | PurS |
| 83 | S | 2.778 | 0.114 | 1.0147438E-13 | 0.022 | 0.041 | 0.068 | PurS |
| 84 | G | 0.975 | 0.000 | 2.88657986E-15 | 0.000 | 0.000 | 0.021 | PurS |
| 85 | V | 0.575 | 0.248 | 0.118892893329 | 0.283 | 0.431 | 0.628 | NE |
| 86 | S | 1.128 | 0.012 | 0 | 0.001 | 0.011 | 0.047 | PurS |
| 87 | E | 0.441 | 0.000 | 9.09517407E-06 | 0.000 | 0.000 | 0.040 | PurS |
| 88 | I | 0.305 | 0.059 | 0.028270025941 | 0.083 | 0.193 | 0.377 | PurS |
| 89 | R | 1.063 | 0.063 | 1.88136173E-10 | 0.023 | 0.059 | 0.120 | PurS |
| 90 | H | 0.678 | 0.181 | 0.042526629602 | 0.155 | 0.267 | 0.425 | PurS |
| 91 | T | 1.820 | 0.070 | 0 | 0.017 | 0.039 | 0.076 | PurS |
| 92 | A | 1.149 | 0.390 | 0.000526363604 | 0.224 | 0.340 | 0.497 | PurS |
| 93 | D | 0.896 | 0.054 | 1.85856659E-05 | 0.024 | 0.061 | 0.124 | PurS |
| 94 | R | 0.463 | 0.285 | 0.375464221196 | 0.394 | 0.615 | 0.918 | NE |
| 95 | W | 5.150 | 0.022 | 0.006791799639 | 0.001 | 0.004 | 0.013 | PurS |
| 96 | R | 2.305 | 0.027 | 0 | 0.003 | 0.012 | 0.030 | PurS |
| 97 | V | 0.874 | 0.067 | 1.52113721E-08 | 0.030 | 0.076 | 0.154 | PurS |
| 98 | S | 0.773 | 0.125 | 4.13776113E-05 | 0.086 | 0.162 | 0.276 | PurS |
| 99 | L | 0.508 | 0.014 | 2.08481177E-08 | 0.002 | 0.027 | 0.118 | PurS |
| 100 | D | 1.174 | 0.027 | 1.11022302E-15 | 0.006 | 0.023 | 0.060 | PurS |
| 101 | V | 0.548 | 0.044 | 3.73651071E-06 | 0.025 | 0.081 | 0.189 | PurS |
| 102 | N | 0.781 | 0.072 | 7.55933462E-05 | 0.044 | 0.093 | 0.168 | PurS |
| 103 | H | 0.945 | 0.067 | 9.68045743E-06 | 0.030 | 0.071 | 0.138 | PurS |
| 104 | F | 1.261 | 0.027 | 1.387779E-14 | 0.005 | 0.021 | 0.054 | PurS |
| 105 | A | 0.554 | 0.132 | 0.000359391061 | 0.119 | 0.239 | 0.423 | PurS |
| 106 | P | 0.947 | 0.012 | 6.66133815E-16 | 0.001 | 0.013 | 0.057 | PurS |
| 107 | D | 0.713 | 0.056 | 0.000160824331 | 0.031 | 0.079 | 0.161 | PurS |
| 108 | E | 1.019 | 0.039 | 1.25656575E-06 | 0.012 | 0.039 | 0.090 | PurS |
| 109 | L | 0.461 | 0.028 | 1.66298819E-06 | 0.010 | 0.061 | 0.189 | PurS |
| 110 | T | 1.018 | 0.184 | 6.79442861E-07 | 0.105 | 0.180 | 0.286 | PurS |

| | | | | | | | | |
|---|---|---|---|---|---|---|---|---|
| 111 | V | 1.137 | 0.090 | 1.22068022E-10 | 0.036 | 0.079 | 0.148 | PurS |
| 112 | K | 1.030 | 0.024 | 2.07236939E-09 | 0.006 | 0.024 | 0.062 | PurS |
| 113 | T | 0.723 | 0.031 | 2.89776447E-09 | 0.011 | 0.042 | 0.111 | PurS |
| 114 | K | 0.921 | 0.050 | 6.89449946E-07 | 0.022 | 0.054 | 0.111 | PurS |
| 115 | D | 1.026 | 0.132 | 9.09826037E-09 | 0.072 | 0.128 | 0.209 | PurS |
| 116 | G | 1.295 | 0.085 | 3.175238E-14 | 0.030 | 0.065 | 0.123 | PurS |
| 117 | V | 0.737 | 0.136 | 0.000199960146 | 0.098 | 0.185 | 0.314 | PurS |
| 118 | V | 0.286 | 0.121 | 0.11372619449 | 0.211 | 0.423 | 0.744 | NE |
| 119 | E | 0.983 | 0.027 | 4.59877692E-11 | 0.007 | 0.028 | 0.072 | PurS |
| 120 | I | 1.830 | 0.017 | 4.4408921E-16 | 0.002 | 0.009 | 0.029 | PurS |
| 121 | T | 1.262 | 0.183 | 3.68111097E-11 | 0.086 | 0.145 | 0.228 | PurS |
| 122 | G | 0.748 | 0.011 | 1.37302392E-11 | 0.001 | 0.014 | 0.062 | PurS |
| 123 | K | 1.278 | 0.048 | 4.763967E-13 | 0.015 | 0.037 | 0.076 | PurS |
| 124 | H | 1.100 | 0.057 | 2.11042295E-12 | 0.021 | 0.052 | 0.106 | PurS |
| 125 | E | 0.780 | 0.059 | 4.35980957E-08 | 0.030 | 0.076 | 0.155 | PurS |
| 126 | E | 1.871 | 0.037 | 1.82531767E-12 | 0.006 | 0.020 | 0.046 | PurS |
| 127 | R | 1.226 | 0.054 | 1.11022302E-16 | 0.017 | 0.044 | 0.090 | PurS |
| 128 | Q | 1.362 | 0.100 | 1.77443911E-08 | 0.037 | 0.074 | 0.130 | PurS |
| 129 | D | 1.856 | 0.037 | 0 | 0.006 | 0.020 | 0.047 | PurS |
| 130 | E | 2.047 | 0.056 | 3.33066907E-16 | 0.011 | 0.027 | 0.056 | PurS |
| 131 | H | 1.770 | 0.036 | 0 | 0.006 | 0.020 | 0.048 | PurS |
| 132 | G | 0.897 | 0.055 | 1.15409904E-11 | 0.022 | 0.061 | 0.133 | PurS |
| 133 | Y | 0.906 | 0.191 | 0.000280120727 | 0.130 | 0.210 | 0.319 | PurS |
| 134 | I | 1.228 | 0.121 | 6.8576661E-09 | 0.054 | 0.098 | 0.163 | PurS |
| 135 | S | 0.961 | 0.022 | 1.7630342E-13 | 0.004 | 0.023 | 0.072 | PurS |
| 136 | R | 0.796 | 0.029 | 1.3933299E-13 | 0.009 | 0.037 | 0.097 | PurS |
| 137 | C | 1.502 | 0.124 | 5.81739795E-08 | 0.045 | 0.083 | 0.137 | PurS |
| 138 | F | 0.860 | 0.040 | 3.16429542E-05 | 0.015 | 0.047 | 0.105 | PurS |
| 139 | T | 0.658 | 0.041 | 6.18397167E-09 | 0.019 | 0.062 | 0.146 | PurS |
| 140 | R | 0.890 | 0.010 | 2.22044605E-16 | 0.001 | 0.012 | 0.051 | PurS |
| 141 | K | 0.284 | 0.032 | 0.000140817226 | 0.034 | 0.111 | 0.259 | PurS |
| 142 | Y | 0.884 | 0.029 | 6.0100902E-07 | 0.008 | 0.033 | 0.087 | PurS |
| 143 | T | 0.705 | 0.114 | 1.91589537E-06 | 0.083 | 0.162 | 0.280 | PurS |
| 144 | L | 0.817 | 0.000 | 2.88658E-14 | 0.000 | 0.000 | 0.031 | PurS |
| 145 | P | 0.799 | 0.000 | 4.9904525E-13 | 0.000 | 0.000 | 0.032 | PurS |
| 146 | P | 1.109 | 0.198 | 6.76920608E-09 | 0.100 | 0.178 | 0.292 | PurS |
| 147 | G | 0.370 | 0.162 | 0.026477519708 | 0.245 | 0.439 | 0.717 | PurS |
| 148 | V | 0.804 | 0.103 | 1.05824418E-08 | 0.061 | 0.128 | 0.232 | PurS |
| 149 | D | 2.022 | 0.128 | 1.71351822E-12 | 0.035 | 0.063 | 0.103 | PurS |
| 150 | P | 0.789 | 0.287 | 0.001728621739 | 0.228 | 0.363 | 0.548 | PurS |

| | | | | | | | | |
|---|---|---|---|---|---|---|---|---|
| 151 | T | 1.495 | 0.124 | 2.19799734E-11 | 0.044 | 0.083 | 0.141 | PurS |
| 152 | Q | 0.712 | 0.261 | 0.006210356224 | 0.231 | 0.367 | 0.552 | PurS |
| 153 | V | 0.915 | 0.041 | 4.51310656E-10 | 0.014 | 0.045 | 0.105 | PurS |
| 154 | S | 1.348 | 0.275 | 2.77650098E-06 | 0.128 | 0.204 | 0.306 | PurS |
| 155 | S | 0.588 | 0.034 | 1.28198166E-07 | 0.014 | 0.057 | 0.149 | PurS |
| 156 | S | 1.123 | 0.070 | 8.11473111E-12 | 0.025 | 0.062 | 0.128 | PurS |
| 157 | L | 0.653 | 0.026 | 9.64291869E-10 | 0.007 | 0.040 | 0.124 | PurS |
| 158 | S | 0.679 | 0.011 | 2.01605399E-12 | 0.001 | 0.016 | 0.072 | PurS |
| 159 | P | 0.622 | 0.000 | 8.237855E-14 | 0.000 | 0.000 | 0.037 | PurS |
| 160 | E | 1.747 | 0.069 | 1.8109958E-12 | 0.017 | 0.040 | 0.077 | PurS |
| 161 | G | 1.438 | 0.022 | 0 | 0.003 | 0.016 | 0.048 | PurS |
| 162 | T | 1.364 | 0.220 | 2.70753716E-08 | 0.098 | 0.162 | 0.248 | PurS |
| 163 | L | 0.644 | 0.013 | 2.72712297E-10 | 0.001 | 0.020 | 0.089 | PurS |
| 164 | T | 1.262 | 0.030 | 0 | 0.006 | 0.024 | 0.061 | PurS |
| 165 | V | 0.562 | 0.043 | 5.63820231E-07 | 0.024 | 0.077 | 0.181 | PurS |
| 166 | E | 1.155 | 0.029 | 2.71681566E-11 | 0.006 | 0.025 | 0.065 | PurS |
| 167 | A | 1.087 | 0.048 | 7.305268E-14 | 0.014 | 0.044 | 0.104 | PurS |
| 168 | P | 1.149 | 0.037 | 6.66133815E-16 | 0.008 | 0.032 | 0.084 | PurS |
| 169 | M | 1.001 | 0.201 | 2.48911689E-05 | 0.116 | 0.201 | 0.320 | PurS |
| 170 | P | 0.375 | 0.150 | 0.073742129403 | 0.213 | 0.400 | 0.678 | PurS |
| 171 | K | 1.156 | 0.098 | 1.59581484E-08 | 0.044 | 0.085 | 0.147 | PurS |
| 172 | L | 0.925 | 0.144 | 1.57173417E-08 | 0.080 | 0.155 | 0.270 | PurS |
| 173 | A | 0.419 | 0.087 | 0.001972175722 | 0.088 | 0.207 | 0.403 | PurS |
| 174 | T | 1.659 | 0.081 | 1.187939E-14 | 0.023 | 0.049 | 0.089 | PurS |
| 175 | Q | 0.966 | 0.087 | 2.60051407E-05 | 0.041 | 0.090 | 0.168 | PurS |
| 176 | S | 1.319 | 0.174 | 1.07174714E-10 | 0.073 | 0.132 | 0.218 | PurS |
| 177 | N | 1.065 | 0.243 | 1.34891162E-06 | 0.134 | 0.229 | 0.359 | PurS |
| 178 | E | 1.657 | 0.020 | 3.308465E-14 | 0.002 | 0.012 | 0.037 | PurS |
| 179 | I | 0.678 | 0.088 | 0.000169727827 | 0.062 | 0.130 | 0.236 | PurS |
| 180 | T | 1.235 | 0.087 | 6.82254253E-12 | 0.032 | 0.071 | 0.132 | PurS |
| 181 | I | 1.271 | 0.009 | 6.66133815E-15 | 0.000 | 0.007 | 0.032 | PurS |
| 182 | P | 1.025 | 0.000 | 0 | 0.000 | 0.000 | 0.023 | PurS |
| 183 | V | 0.933 | 0.078 | 2.60439936E-09 | 0.036 | 0.083 | 0.162 | PurS |
| 184 | T | 0.937 | 0.099 | 1.49723932E-07 | 0.053 | 0.105 | 0.187 | PurS |
| 185 | F | 1.183 | 0.194 | 1.15141023E-06 | 0.099 | 0.164 | 0.253 | PurS |
| 186 | E | 1.088 | 0.200 | 0.001631215619 | 0.111 | 0.184 | 0.283 | PurS |
| 187 | S | 1.823 | 0.433 | 1.75729765E-05 | 0.159 | 0.238 | 0.343 | PurS |
| 188 | R | 1.116 | 0.271 | 1.36447903E-05 | 0.151 | 0.243 | 0.371 | PurS |
| 189 | A | 0.536 | 0.234 | 0.04046742205 | 0.241 | 0.436 | 0.716 | PurS |
| 190 | Q | 2.223 | 0.209 | 1.69819389E-07 | 0.052 | 0.094 | 0.155 | PurS |

| | | | | | | | | |
|---|---|---|---|---|---|---|---|---|
| 191 | L | 0.852 | 0.267 | 0.001371257116 | 0.189 | 0.313 | 0.490 | PurS |
| 192 | G | 1.374 | 0.267 | 0.00013436435 | 0.105 | 0.195 | 0.331 | PurS |
| 193 | G | 0.654 | 0.187 | 0.006046262797 | 0.139 | 0.286 | 0.526 | PurS |
| 194 | P | 0.924 | 0.427 | 0.034146336006 | 0.263 | 0.463 | 0.760 | PurS |
| 195 | E | 0.596 | 0.186 | 0.029479244209 | 0.164 | 0.313 | 0.543 | PurS |
| 196 | A | 0.768 | 0.320 | 0.032964715506 | 0.225 | 0.417 | 0.714 | PurS |
| 197 | A | 1.190 | 0.295 | 0.001497372455 | 0.146 | 0.248 | 0.395 | PurS |
| 198 | K | 1.221 | 0.056 | 7.66397849E-08 | 0.016 | 0.046 | 0.099 | PurS |
| 199 | S | 2.577 | 0.475 | 3.75238482E-06 | 0.115 | 0.184 | 0.284 | PurS |
| 200 | D | 1.674 | 0.245 | 1.83375631E-05 | 0.088 | 0.146 | 0.229 | PurS |
| 201 | E | 2.754 | 0.383 | 2.75384718E-05 | 0.088 | 0.139 | 0.210 | PurS |
| 202 | T | 0.638 | 0.538 | 0.625520352342 | 0.547 | 0.844 | 1.256 | NE |
| 203 | A | 0.577 | 0.516 | 0.734620768704 | 0.611 | 0.894 | 1.265 | NE |
| 204 | A | 0.693 | 0.326 | 0.097774997511 | 0.311 | 0.470 | 0.680 | PurS |
| 205 | K | 1.081 | 0.117 | 2.95478418E-06 | 0.059 | 0.109 | 0.182 | PurS |

**HspB3**

| site | amino acid | alpha | beta | p-value | dN/dS LB | dN/dS MLE (omega) | dN/dS UB | selection |
|---|---|---|---|---|---|---|---|---|
| 1 | M | 0.000 | 0.000 | n/a | n/a | n/a | n/a | ND † |
| 2 | A | 0.689 | 0.132 | 0.000604368921 | 0.082 | 0.191 | 0.374 | PurS |
| 3 | K | 0.318 | 0.414 | 0.612465590328 | 0.802 | 1.299 | 1.981 | NE |
| 4 | I | 0.555 | 0.268 | 0.104829502074 | 0.263 | 0.483 | 0.806 | NE |
| 5 | I | 1.164 | 0.282 | 0.000161371795 | 0.138 | 0.242 | 0.393 | PurS |
| 6 | L | 1.360 | 0.179 | 6.87384819E-07 | 0.062 | 0.132 | 0.241 | PurS |
| 7 | R | 0.947 | 0.118 | 4.94398965E-06 | 0.053 | 0.124 | 0.243 | PurS |
| 8 | H | 0.250 | 0.000 | 0.000789160946 | 0.000 | 0.000 | 0.113 | PurS |
| 9 | L | 0.613 | 0.224 | 0.070153040544 | 0.184 | 0.365 | 0.639 | PurS |
| 10 | I | 1.380 | 0.105 | 1.31204374E-07 | 0.030 | 0.076 | 0.157 | PurS |
| 11 | E | 0.251 | 0.312 | 0.713834658029 | 0.710 | 1.246 | 2.017 | NE |
| 12 | I | 0.942 | 0.108 | 4.78569243E-07 | 0.049 | 0.115 | 0.224 | PurS |
| 13 | P | 1.105 | 0.000 | 4.196643E-14 | 0.000 | 0.000 | 0.026 | PurS |
| 14 | V | 0.872 | 0.089 | 3.60229327E-06 | 0.032 | 0.102 | 0.241 | PurS |
| 15 | R | 1.353 | 0.000 | 2.33146835E-15 | 0.000 | 0.000 | 0.024 | PurS |
| 16 | Y | 1.399 | 0.065 | 1.49640551E-07 | 0.011 | 0.046 | 0.121 | PurS |
| 17 | Q | 1.101 | 0.100 | 1.17721745E-05 | 0.033 | 0.091 | 0.198 | PurS |
| 18 | E | 0.541 | 0.251 | 0.160980737935 | 0.248 | 0.465 | 0.788 | NE |
| 19 | E | 0.910 | 0.496 | 0.188971598342 | 0.342 | 0.545 | 0.824 | NE |
| 20 | F | 0.778 | 0.073 | 2.39952852E-05 | 0.029 | 0.094 | 0.220 | PurS |
| 21 | E | 0.701 | 0.318 | 0.073818609676 | 0.260 | 0.454 | 0.737 | PurS |

| | | | | | | | | |
|---|---|---|---|---|---|---|---|---|
| 22 | A | 0.837 | 0.581 | 0.303171898584 | 0.452 | 0.694 | 1.024 | NE |
| 23 | R | 0.959 | 0.238 | 0.000257886929 | 0.127 | 0.249 | 0.434 | PurS |
| 24 | G | 0.884 | 0.210 | 0.000964616715 | 0.122 | 0.237 | 0.413 | PurS |
| 25 | L | 1.278 | 0.049 | 2.66621725E-10 | 0.006 | 0.039 | 0.121 | PurS |
| 26 | E | 0.825 | 0.256 | 0.012887363317 | 0.165 | 0.310 | 0.527 | PurS |
| 27 | D | 0.954 | 0.292 | 0.0078842497 | 0.174 | 0.306 | 0.496 | PurS |
| 28 | C | 0.459 | 0.146 | 0.062670691971 | 0.153 | 0.319 | 0.579 | PurS |
| 29 | R | 1.765 | 0.170 | 1.62597735E-09 | 0.049 | 0.096 | 0.176 | PurS |
| 30 | L | 0.319 | 0.073 | 0.018240172043 | 0.057 | 0.229 | 0.601 | PurS |
| 31 | D | 1.122 | 0.128 | 8.40506444E-06 | 0.049 | 0.114 | 0.223 | PurS |
| 32 | H | 2.367 | 0.000 | 0 | 0.000 | 0.000 | 0.013 | PurS |
| 33 | A | 1.044 | 0.510 | 0.039376172643 | 0.314 | 0.488 | 0.723 | PurS |
| 34 | L | 1.954 | 0.027 | 5.77315973E-15 | 0.001 | 0.014 | 0.060 | PurS |
| 35 | Y | 2.922 | 0.081 | 1.0336176E-13 | 0.009 | 0.028 | 0.065 | PurS |
| 36 | A | 1.349 | 0.017 | 1.776357E-14 | 0.001 | 0.012 | 0.054 | PurS |
| 37 | L | 0.295 | 0.000 | 0.000135645509 | 0.000 | 0.000 | 0.167 | PurS |
| 38 | P | 3.177 | 0.000 | 0 | 0.000 | 0.000 | 0.009 | PurS |
| 39 | G | 0.957 | 0.018 | 1.22309385E-10 | 0.001 | 0.019 | 0.084 | PurS |
| 40 | P | 0.571 | 0.031 | 1.04264593E-06 | 0.009 | 0.054 | 0.166 | PurS |
| 41 | T | 0.742 | 0.362 | 0.050813387752 | 0.300 | 0.488 | 0.749 | PurS |
| 42 | I | 1.721 | 0.571 | 0.000857468052 | 0.217 | 0.332 | 0.485 | PurS |
| 43 | V | 0.698 | 0.702 | 0.990204788325 | 0.650 | 1.005 | 1.503 | NE |
| 44 | D | 1.566 | 0.312 | 0.000158916671 | 0.114 | 0.200 | 0.323 | PurS |
| 45 | L | 0.489 | 0.665 | 0.420895025702 | 0.838 | 1.361 | 2.097 | NE |
| 46 | R | 1.040 | 0.599 | 0.145833982122 | 0.392 | 0.577 | 0.824 | NE |
| 47 | K | 0.725 | 0.593 | 0.632719834012 | 0.534 | 0.818 | 1.193 | NE |
| 48 | T | 0.352 | 1.068 | 0.009985701289 | 2.132 | 3.036 | 4.231 | DivS |
| 49 | R | 1.248 | 0.530 | 0.026246280351 | 0.272 | 0.424 | 0.634 | PurS |
| 50 | A | 2.692 | 0.774 | 0.001895349571 | 0.173 | 0.287 | 0.456 | PurS |
| 51 | A | 2.003 | 0.944 | 0.016864443741 | 0.318 | 0.471 | 0.676 | PurS |
| 52 | Q | 1.335 | 1.139 | 0.69842988751 | 0.578 | 0.853 | 1.238 | NE |
| 53 | S | 0.771 | 1.276 | 0.184575667936 | 1.144 | 1.654 | 2.349 | NE |
| 54 | P | 1.633 | 0.750 | 0.022293995931 | 0.307 | 0.460 | 0.666 | PurS |
| 55 | P | 3.108 | 0.803 | 1.4133865E-05 | 0.174 | 0.258 | 0.375 | PurS |
| 56 | V | 1.300 | 1.129 | 0.679404950917 | 0.580 | 0.869 | 1.271 | NE |
| 57 | D | 1.218 | 0.741 | 0.288474071244 | 0.406 | 0.608 | 0.883 | NE |
| 58 | S | 1.234 | 0.712 | 0.093031074309 | 0.376 | 0.577 | 0.855 | PurS |
| 59 | A | 3.773 | 0.813 | 2.00571916E-06 | 0.145 | 0.215 | 0.314 | PurS |
| 60 | A | 1.777 | 1.078 | 0.138704535665 | 0.412 | 0.607 | 0.870 | NE |
| 61 | E | 1.640 | 1.085 | 0.315840781086 | 0.451 | 0.662 | 0.943 | NE |

| | | | | | | | | |
|---|---|---|---|---|---|---|---|---|
| 62 | T | 1.866 | 1.286 | 0.341366907618 | 0.491 | 0.689 | 0.952 | NE |
| 63 | P | 1.084 | 0.841 | 0.459303291898 | 0.540 | 0.775 | 1.087 | NE |
| 64 | P | 1.775 | 0.630 | 0.001390323829 | 0.237 | 0.355 | 0.513 | PurS |
| 65 | R | 1.227 | 1.234 | 1 | 0.706 | 1.005 | 1.401 | NE |
| 66 | E | 0.426 | 0.596 | 0.456166425854 | 0.901 | 1.398 | 2.080 | NE |
| 67 | G | 0.697 | 0.517 | 0.471393601857 | 0.469 | 0.741 | 1.109 | NE |
| 68 | K | 1.713 | 0.521 | 0.002997524504 | 0.197 | 0.304 | 0.450 | PurS |
| 69 | S | 0.816 | 0.606 | 0.381936950151 | 0.499 | 0.743 | 1.073 | NE |
| 70 | H | 0.965 | 0.831 | 0.659105509567 | 0.596 | 0.862 | 1.210 | NE |
| 71 | F | 1.408 | 0.035 | 2.05962247E-10 | 0.004 | 0.025 | 0.076 | PurS |
| 72 | Q | 0.897 | 0.126 | 0.000178768458 | 0.056 | 0.141 | 0.289 | PurS |
| 73 | I | 0.513 | 0.208 | 0.066748790672 | 0.202 | 0.405 | 0.723 | PurS |
| 74 | L | 0.792 | 0.025 | 8.79983665E-08 | 0.002 | 0.031 | 0.140 | PurS |
| 75 | L | 0.406 | 0.078 | 0.004346474521 | 0.048 | 0.193 | 0.508 | PurS |
| 76 | D | 4.885 | 0.000 | 0 | 0.000 | 0.000 | 0.007 | PurS |
| 77 | V | 0.594 | 0.044 | 3.04429972E-05 | 0.012 | 0.074 | 0.230 | PurS |
| 78 | V | 0.497 | 0.043 | 0.000103815306 | 0.014 | 0.086 | 0.267 | PurS |
| 79 | Q | 0.536 | 0.000 | 8.49549562E-06 | 0.000 | 0.000 | 0.071 | PurS |
| 80 | F | 1.353 | 0.000 | 1.10436771E-10 | 0.000 | 0.000 | 0.023 | PurS |
| 81 | L | 0.816 | 0.116 | 6.08778326E-05 | 0.056 | 0.143 | 0.293 | PurS |
| 82 | P | 1.849 | 0.016 | 0 | 0.000 | 0.008 | 0.037 | PurS |
| 83 | E | 0.733 | 0.000 | 2.82990966E-08 | 0.000 | 0.000 | 0.048 | PurS |
| 84 | D | 1.632 | 0.035 | 1.07412967E-11 | 0.004 | 0.021 | 0.066 | PurS |
| 85 | I | 0.444 | 0.050 | 0.000527431322 | 0.028 | 0.113 | 0.297 | PurS |
| 86 | I | 0.352 | 0.052 | 0.003904227194 | 0.037 | 0.148 | 0.386 | PurS |
| 87 | I | 0.745 | 0.000 | 3.96889499E-09 | 0.000 | 0.000 | 0.043 | PurS |
| 88 | Q | 0.243 | 0.000 | 0.001879002164 | 0.000 | 0.000 | 0.157 | PurS |
| 89 | T | 0.607 | 0.161 | 0.003533658991 | 0.128 | 0.266 | 0.484 | PurS |
| 90 | F | 4.093 | 0.034 | 0 | 0.001 | 0.008 | 0.025 | PurS |
| 91 | E | 1.353 | 0.000 | 2.34949837E-11 | 0.000 | 0.000 | 0.026 | PurS |
| 92 | G | 0.226 | 0.000 | 0.000253549122 | 0.000 | 0.000 | 0.153 | PurS |
| 93 | W | 0.000 | 0.000 | n/a | n/a | n/a | n/a | ND † |
| 94 | L | 0.420 | 0.000 | 2.01811371E-06 | 0.000 | 0.000 | 0.099 | PurS |
| 95 | L | 0.352 | 0.024 | 0.000533319883 | 0.004 | 0.070 | 0.308 | PurS |
| 96 | I | 1.466 | 0.141 | 1.26751959E-07 | 0.044 | 0.096 | 0.182 | PurS |
| 97 | K | 1.070 | 0.051 | 3.69015244E-08 | 0.012 | 0.048 | 0.124 | PurS |
| 98 | A | 1.044 | 0.088 | 1.89496596E-08 | 0.030 | 0.084 | 0.182 | PurS |
| 99 | Q | 2.131 | 0.122 | 1.48688872E-09 | 0.023 | 0.057 | 0.117 | PurS |
| 100 | H | 4.398 | 0.000 | 0 | 0.000 | 0.000 | 0.007 | PurS |
| 101 | G | 0.674 | 0.079 | 2.24219478E-05 | 0.036 | 0.117 | 0.273 | PurS |

| | | | | | | | | |
|---|---|---|---|---|---|---|---|---|
| 102 | T | 0.409 | 0.315 | 0.55970934933 | 0.455 | 0.771 | 1.215 | NE |
| 103 | R | 0.928 | 0.016 | 9.57686153E-11 | 0.001 | 0.017 | 0.075 | PurS |
| 104 | M | 0.000 | 0.000 | n/a | n/a | n/a | n/a | ND † |
| 105 | D | 5.743 | 0.017 | 0 | 0.000 | 0.003 | 0.013 | PurS |
| 106 | E | 1.336 | 0.018 | 2.04653627E-10 | 0.001 | 0.014 | 0.062 | PurS |
| 107 | H | 2.707 | 0.016 | 7.77156117E-15 | 0.000 | 0.006 | 0.026 | PurS |
| 108 | G | 1.728 | 0.000 | 0 | 0.000 | 0.000 | 0.020 | PurS |
| 109 | F | 1.278 | 0.035 | 1.65641201E-09 | 0.005 | 0.027 | 0.084 | PurS |
| 110 | I | 0.648 | 0.185 | 0.009525652102 | 0.142 | 0.286 | 0.507 | PurS |
| 111 | S | 1.504 | 0.075 | 9.30977517E-12 | 0.016 | 0.050 | 0.118 | PurS |
| 112 | R | 1.353 | 0.000 | 3.763656E-14 | 0.000 | 0.000 | 0.023 | PurS |
| 113 | S | 0.732 | 0.065 | 5.64042755E-05 | 0.032 | 0.089 | 0.192 | PurS |
| 114 | F | 0.920 | 0.016 | 2.84525546E-08 | 0.001 | 0.018 | 0.079 | PurS |
| 115 | T | 0.783 | 0.077 | 8.74003153E-07 | 0.035 | 0.098 | 0.211 | PurS |
| 116 | R | 1.353 | 0.000 | 3.841372E-14 | 0.000 | 0.000 | 0.025 | PurS |
| 117 | Q | 0.916 | 0.060 | 5.20045051E-06 | 0.016 | 0.065 | 0.169 | PurS |
| 118 | Y | 1.713 | 0.000 | 9.3181018E-13 | 0.000 | 0.000 | 0.024 | PurS |
| 119 | K | 0.957 | 0.141 | 6.866416E-05 | 0.067 | 0.147 | 0.278 | PurS |
| 120 | L | 0.606 | 0.000 | 9.30834724E-08 | 0.000 | 0.000 | 0.082 | PurS |
| 121 | P | 0.950 | 0.016 | 2.82710522E-11 | 0.001 | 0.016 | 0.071 | PurS |
| 122 | D | 1.434 | 0.361 | 0.00076493091 | 0.152 | 0.252 | 0.392 | PurS |
| 123 | G | 1.103 | 0.144 | 1.7225175E-06 | 0.055 | 0.130 | 0.254 | PurS |
| 124 | V | 1.700 | 0.463 | 0.000109566223 | 0.164 | 0.273 | 0.424 | PurS |
| 125 | E | 1.123 | 0.307 | 0.002227759672 | 0.152 | 0.273 | 0.451 | PurS |
| 126 | I | 0.592 | 0.224 | 0.023644993733 | 0.202 | 0.379 | 0.640 | PurS |
| 127 | K | 0.799 | 0.195 | 0.002182780492 | 0.126 | 0.244 | 0.423 | PurS |
| 128 | D | 0.652 | 0.017 | 4.06735951E-06 | 0.002 | 0.027 | 0.118 | PurS |
| 129 | L | 0.800 | 0.026 | 3.16855646E-07 | 0.002 | 0.033 | 0.145 | PurS |
| 130 | S | 1.058 | 0.178 | 1.14180942E-05 | 0.084 | 0.168 | 0.298 | PurS |
| 131 | A | 1.623 | 0.000 | 0 | 0.000 | 0.000 | 0.020 | PurS |
| 132 | V | 0.453 | 0.503 | 0.809258067078 | 0.716 | 1.110 | 1.653 | NE |
| 133 | L | 0.272 | 0.113 | 0.14145130318 | 0.164 | 0.415 | 0.848 | NE |
| 134 | C | 1.353 | 0.000 | 1.26976207E-12 | 0.000 | 0.000 | 0.022 | PurS |
| 135 | H | 2.167 | 0.000 | 3.33066907E-16 | 0.000 | 0.000 | 0.014 | PurS |
| 136 | D | 0.923 | 0.000 | 3.78672538E-10 | 0.000 | 0.000 | 0.038 | PurS |
| 137 | G | 0.763 | 0.000 | 2.21890284E-10 | 0.000 | 0.000 | 0.046 | PurS |
| 138 | I | 0.506 | 0.034 | 5.16866082E-05 | 0.011 | 0.067 | 0.208 | PurS |
| 139 | L | 0.580 | 0.000 | 2.79329044E-07 | 0.000 | 0.000 | 0.088 | PurS |
| 140 | V | 0.312 | 0.045 | 0.008807843429 | 0.024 | 0.143 | 0.445 | PurS |
| 141 | V | 0.272 | 0.046 | 0.024389870717 | 0.028 | 0.169 | 0.523 | PurS |

| | | | | | | | | |
|---|---|---|---|---|---|---|---|---|
| 142 | E | 0.597 | 0.097 | 0.001907578245 | 0.058 | 0.163 | 0.355 | PurS |
| 143 | V | 1.767 | 0.431 | 6.66135701E-05 | 0.151 | 0.244 | 0.374 | PurS |
| 144 | K | 0.797 | 0.099 | 0.00080450059 | 0.045 | 0.124 | 0.265 | PurS |
| 145 | D | 1.013 | 0.522 | 0.127900266881 | 0.328 | 0.515 | 0.773 | NE |
| 146 | P | 1.496 | 0.538 | 0.002530109727 | 0.226 | 0.360 | 0.544 | PurS |
| 147 | V | 0.982 | 1.727 | 0.090552155467 | 1.241 | 1.760 | 2.460 | DivS |
| 148 | G | 1.176 | 0.452 | 0.007490132708 | 0.233 | 0.384 | 0.603 | PurS |
| 149 | T | 1.457 | 0.670 | 0.018887251506 | 0.309 | 0.460 | 0.662 | PurS |
| 150 | K | 1.064 | 0.816 | 0.535735025809 | 0.517 | 0.766 | 1.101 | NE |

**HspB5**

| site | amino acid | alpha | beta | p-value | dN/dS LB | dN/dS MLE (omega) | dN/dS UB | selection |
|---|---|---|---|---|---|---|---|---|
| 1 | M | 0.000 | 0.000 | n/a | n/a | n/a | n/a | ND † |
| 2 | D | 0.464 | 0.016 | 8.21075889E-05 | 0.002 | 0.034 | 0.152 | PurS |
| 3 | I | 0.759 | 0.094 | 9.80871606E-05 | 0.049 | 0.123 | 0.252 | PurS |
| 4 | A | 0.534 | 0.169 | 0.017231938869 | 0.151 | 0.316 | 0.579 | PurS |
| 5 | I | 1.093 | 0.031 | 2.73704504E-09 | 0.005 | 0.028 | 0.087 | PurS |
| 6 | H | 1.361 | 0.072 | 2.50424667E-06 | 0.016 | 0.053 | 0.124 | PurS |
| 7 | H | 1.080 | 0.207 | 0.000420765143 | 0.102 | 0.191 | 0.326 | PurS |
| 8 | P | 1.039 | 0.000 | 3.27948779E-12 | 0.000 | 0.000 | 0.033 | PurS |
| 9 | W | 1.147 | 0.038 | 0.000181711035 | 0.006 | 0.033 | 0.104 | PurS |
| 10 | I | 1.006 | 0.208 | 0.001875643147 | 0.111 | 0.207 | 0.351 | PurS |
| 11 | R | 1.500 | 0.000 | 5.55111512E-16 | 0.000 | 0.000 | 0.023 | PurS |
| 12 | R | 1.476 | 0.017 | 1.0869083E-13 | 0.001 | 0.012 | 0.051 | PurS |
| 13 | P | 0.832 | 0.098 | 2.48650688E-05 | 0.042 | 0.118 | 0.255 | PurS |
| 14 | F | 1.604 | 0.220 | 4.03274361E-06 | 0.075 | 0.137 | 0.229 | PurS |
| 15 | F | 2.020 | 0.309 | 1.12415807E-05 | 0.089 | 0.153 | 0.244 | PurS |
| 16 | P | 0.796 | 0.163 | 0.000189981263 | 0.093 | 0.204 | 0.383 | PurS |
| 17 | F | 1.519 | 0.211 | 3.76013871E-05 | 0.072 | 0.139 | 0.241 | PurS |
| 18 | H | 0.633 | 0.330 | 0.217278343468 | 0.306 | 0.521 | 0.830 | NE |
| 19 | S | 1.540 | 0.253 | 3.84869745E-06 | 0.090 | 0.165 | 0.275 | PurS |
| 20 | P | 1.353 | 0.000 | 6.8922645E-13 | 0.000 | 0.000 | 0.026 | PurS |
| 21 | S | 1.158 | 0.178 | 0.00015073032 | 0.079 | 0.154 | 0.266 | PurS |
| 22 | R | 1.125 | 0.018 | 8.88344953E-12 | 0.001 | 0.016 | 0.072 | PurS |
| 23 | L | 0.054 | 0.083 | 0.682005645602 | 0.546 | 1.535 | 3.328 | NE |
| 24 | F | 0.751 | 0.073 | 9.01644335E-06 | 0.035 | 0.098 | 0.212 | PurS |
| 25 | D | 0.838 | 0.117 | 0.0011042174 | 0.059 | 0.139 | 0.272 | PurS |
| 26 | Q | 0.661 | 0.041 | 0.001080809961 | 0.010 | 0.062 | 0.192 | PurS |
| 27 | F | 0.833 | 0.307 | 0.026922261286 | 0.217 | 0.368 | 0.583 | PurS |

| | | | | | | | | |
|---|---|---|---|---|---|---|---|---|
| 28 | F | 8.155 | 0.028 | 0 | 0.001 | 0.003 | 0.011 | PurS |
| 29 | G | 1.163 | 0.018 | 8.089085E-13 | 0.001 | 0.016 | 0.069 | PurS |
| 30 | E | 2.028 | 0.033 | 1.64837033E-11 | 0.003 | 0.016 | 0.050 | PurS |
| 31 | H | 1.219 | 0.104 | 1.78364489E-05 | 0.034 | 0.085 | 0.176 | PurS |
| 32 | L | 0.712 | 0.105 | 9.98523419E-05 | 0.052 | 0.147 | 0.321 | PurS |
| 33 | L | 1.826 | 0.397 | 4.55438597E-05 | 0.123 | 0.217 | 0.357 | PurS |
| 34 | E | 0.957 | 0.051 | 5.30246273E-05 | 0.013 | 0.053 | 0.140 | PurS |
| 35 | S | 0.864 | 0.288 | 0.003323789063 | 0.193 | 0.334 | 0.535 | PurS |
| 36 | D | 1.387 | 0.159 | 1.53603568E-05 | 0.055 | 0.115 | 0.210 | PurS |
| 37 | L | 0.684 | 0.173 | 0.002662861337 | 0.114 | 0.253 | 0.477 | PurS |
| 38 | F | 1.473 | 0.104 | 4.63674167E-07 | 0.028 | 0.071 | 0.144 | PurS |
| 39 | P | 1.471 | 0.170 | 1.08379488E-08 | 0.053 | 0.115 | 0.215 | PurS |
| 40 | T | 1.062 | 0.389 | 0.004334959966 | 0.221 | 0.366 | 0.573 | PurS |
| 41 | S | 1.052 | 0.159 | 5.06686015E-05 | 0.072 | 0.151 | 0.278 | PurS |
| 42 | T | 0.846 | 0.291 | 0.010596206855 | 0.190 | 0.344 | 0.577 | PurS |
| 43 | S | 1.077 | 0.337 | 0.010208985255 | 0.179 | 0.313 | 0.509 | PurS |
| 44 | L | 0.766 | 0.372 | 0.138549925529 | 0.244 | 0.485 | 0.872 | NE |
| 45 | S | 2.862 | 0.202 | 3.71392702E-08 | 0.034 | 0.071 | 0.129 | PurS |
| 46 | P | 0.940 | 0.162 | 5.09464412E-05 | 0.078 | 0.172 | 0.326 | PurS |
| 47 | F | 0.362 | 0.016 | 0.001554472845 | 0.003 | 0.043 | 0.197 | PurS |
| 48 | Y | 0.800 | 0.193 | 0.005884140756 | 0.120 | 0.241 | 0.430 | PurS |
| 49 | L | 1.049 | 0.432 | 0.018328370171 | 0.257 | 0.411 | 0.623 | PurS |
| 50 | R | 0.632 | 0.018 | 9.14338222E-08 | 0.002 | 0.029 | 0.128 | PurS |
| 51 | P | 1.407 | 0.439 | 0.000612766607 | 0.194 | 0.312 | 0.476 | PurS |
| 52 | P | 1.110 | 0.196 | 7.13337976E-05 | 0.088 | 0.177 | 0.314 | PurS |
| 53 | S | 3.430 | 0.059 | 4.97231922E-10 | 0.001 | 0.017 | 0.077 | PurS |
| 54 | F | 1.878 | 0.152 | 4.34475695E-07 | 0.040 | 0.081 | 0.144 | PurS |
| 55 | L | 1.305 | 0.303 | 0.000125170903 | 0.128 | 0.232 | 0.383 | PurS |
| 56 | R | 0.879 | 0.035 | 6.98992065E-08 | 0.007 | 0.040 | 0.125 | PurS |
| 57 | A | 0.695 | 0.647 | 0.879723022536 | 0.619 | 0.931 | 1.351 | NE |
| 58 | P | 0.840 | 0.136 | 6.53702061E-05 | 0.069 | 0.162 | 0.317 | PurS |
| 59 | S | 0.358 | 0.159 | 0.213991505771 | 0.229 | 0.444 | 0.769 | NE |
| 60 | W | 10000.000 | 0.035 | 0.589362284229 | 0.000 | 0.000 | 0.000 | ND † |
| 61 | F | 0.830 | 0.469 | 0.116165476564 | 0.352 | 0.565 | 0.858 | NE |
| 62 | D | 0.567 | 0.153 | 0.045666086379 | 0.129 | 0.270 | 0.496 | PurS |
| 63 | T | 1.024 | 0.204 | 7.56178627E-05 | 0.106 | 0.199 | 0.339 | PurS |
| 64 | G | 0.901 | 0.072 | 1.49412233E-07 | 0.025 | 0.080 | 0.186 | PurS |
| 65 | L | 0.620 | 0.139 | 0.002768308987 | 0.095 | 0.223 | 0.439 | PurS |
| 66 | S | 0.944 | 0.059 | 1.85367415E-08 | 0.016 | 0.063 | 0.164 | PurS |
| 67 | E | 0.240 | 0.000 | 0.003876398487 | 0.000 | 0.000 | 0.132 | PurS |

| | | | | | | | | |
|---|---|---|---|---|---|---|---|---|
| 68 | M | 1.792 | 0.037 | 0.002361045351 | 0.005 | 0.021 | 0.054 | PurS |
| 69 | R | 0.926 | 0.077 | 1.87041193E-07 | 0.026 | 0.084 | 0.197 | PurS |
| 70 | L | 0.331 | 0.262 | 0.656399187045 | 0.429 | 0.791 | 1.335 | NE |
| 71 | E | 0.882 | 0.230 | 0.019650472382 | 0.142 | 0.260 | 0.436 | PurS |
| 72 | K | 1.009 | 0.047 | 4.36748697E-06 | 0.012 | 0.047 | 0.122 | PurS |
| 73 | D | 0.414 | 0.016 | 0.000419719631 | 0.002 | 0.039 | 0.171 | PurS |
| 74 | R | 1.720 | 0.117 | 2.28627517E-09 | 0.029 | 0.068 | 0.133 | PurS |
| 75 | F | 1.161 | 0.014 | 3.20600213E-11 | 0.001 | 0.012 | 0.054 | PurS |
| 76 | S | 1.408 | 0.245 | 2.3776409E-06 | 0.093 | 0.174 | 0.295 | PurS |
| 77 | V | 0.702 | 0.068 | 4.94338164E-06 | 0.030 | 0.097 | 0.228 | PurS |
| 78 | N | 1.454 | 0.093 | 1.21806516E-08 | 0.025 | 0.064 | 0.130 | PurS |
| 79 | L | 0.786 | 0.044 | 4.71791441E-07 | 0.009 | 0.056 | 0.174 | PurS |
| 80 | D | 0.861 | 0.000 | 2.20662599E-10 | 0.000 | 0.000 | 0.035 | PurS |
| 81 | V | 0.492 | 0.019 | 2.16197208E-05 | 0.002 | 0.038 | 0.167 | PurS |
| 82 | K | 1.616 | 0.000 | 1.0905854E-10 | 0.000 | 0.000 | 0.018 | PurS |
| 83 | H | 0.902 | 0.049 | 5.2508809E-07 | 0.013 | 0.054 | 0.142 | PurS |
| 84 | F | 0.422 | 0.000 | 1.5374534E-05 | 0.000 | 0.000 | 0.064 | PurS |
| 85 | S | 0.879 | 0.123 | 2.05839117E-05 | 0.060 | 0.140 | 0.274 | PurS |
| 86 | P | 1.068 | 0.018 | 6.09379214E-12 | 0.001 | 0.017 | 0.075 | PurS |
| 87 | E | 1.180 | 0.033 | 2.11013845E-07 | 0.005 | 0.028 | 0.087 | PurS |
| 88 | E | 3.036 | 0.065 | 3.44169138E-15 | 0.007 | 0.022 | 0.051 | PurS |
| 89 | L | 0.702 | 0.022 | 1.93893333E-07 | 0.002 | 0.032 | 0.141 | PurS |
| 90 | K | 1.377 | 0.226 | 3.12674651E-05 | 0.087 | 0.164 | 0.282 | PurS |
| 91 | V | 0.874 | 0.018 | 2.04778248E-08 | 0.001 | 0.021 | 0.092 | PurS |
| 92 | K | 1.421 | 0.047 | 6.93186425E-08 | 0.008 | 0.033 | 0.085 | PurS |
| 93 | V | 0.652 | 0.091 | 0.000127827728 | 0.050 | 0.140 | 0.305 | PurS |
| 94 | L | 1.915 | 0.398 | 4.41654765E-06 | 0.123 | 0.208 | 0.331 | PurS |
| 95 | G | 1.391 | 0.045 | 3.53927998E-12 | 0.006 | 0.033 | 0.094 | PurS |
| 96 | D | 1.400 | 0.048 | 9.23792709E-10 | 0.009 | 0.035 | 0.090 | PurS |
| 97 | V | 0.640 | 0.165 | 0.019231836998 | 0.129 | 0.258 | 0.456 | PurS |
| 98 | I | 0.422 | 0.106 | 0.010093179334 | 0.107 | 0.252 | 0.492 | PurS |
| 99 | E | 1.451 | 0.066 | 5.45070933E-09 | 0.014 | 0.046 | 0.107 | PurS |
| 100 | V | 1.186 | 0.122 | 1.59181582E-07 | 0.044 | 0.103 | 0.203 | PurS |
| 101 | H | 3.295 | 0.120 | 1.08624221E-12 | 0.016 | 0.036 | 0.071 | PurS |
| 102 | G | 0.995 | 0.054 | 1.87435228E-08 | 0.013 | 0.055 | 0.142 | PurS |
| 103 | K | 0.902 | 0.063 | 2.4515398E-06 | 0.022 | 0.070 | 0.164 | PurS |
| 104 | H | 3.303 | 0.000 | 0 | 0.000 | 0.000 | 0.009 | PurS |
| 105 | E | 1.334 | 0.016 | 9.98378047E-11 | 0.001 | 0.012 | 0.054 | PurS |
| 106 | E | 5.768 | 0.049 | 0 | 0.002 | 0.009 | 0.022 | PurS |
| 107 | R | 0.963 | 0.000 | 5.68256553E-12 | 0.000 | 0.000 | 0.035 | PurS |

| | | | | | | | | |
|---|---|---|---|---|---|---|---|---|
| 108 | Q | 0.000 | 0.000 | n/a | n/a | n/a | n/a | ND † |
| 109 | D | 0.763 | 0.000 | 6.56040444E-10 | 0.000 | 0.000 | 0.040 | PurS |
| 110 | E | 1.183 | 0.049 | 1.80486133E-07 | 0.010 | 0.042 | 0.109 | PurS |
| 111 | H | 1.799 | 0.016 | 1.698641E-14 | 0.001 | 0.009 | 0.040 | PurS |
| 112 | G | 0.966 | 0.000 | 6.79012402E-12 | 0.000 | 0.000 | 0.034 | PurS |
| 113 | F | 1.794 | 0.177 | 6.70386651E-07 | 0.051 | 0.099 | 0.171 | PurS |
| 114 | I | 0.852 | 0.181 | 0.000610186399 | 0.105 | 0.212 | 0.376 | PurS |
| 115 | S | 0.655 | 0.036 | 2.06429938E-06 | 0.009 | 0.055 | 0.171 | PurS |
| 116 | R | 0.874 | 0.000 | 9.39093248E-12 | 0.000 | 0.000 | 0.036 | PurS |
| 117 | E | 1.147 | 0.016 | 4.11984843E-07 | 0.001 | 0.014 | 0.064 | PurS |
| 118 | F | 0.703 | 0.000 | 9.25558301E-08 | 0.000 | 0.000 | 0.039 | PurS |
| 119 | H | 1.178 | 0.355 | 0.009477465438 | 0.179 | 0.301 | 0.474 | PurS |
| 120 | R | 0.877 | 0.000 | 3.98162614E-11 | 0.000 | 0.000 | 0.033 | PurS |
| 121 | K | 1.161 | 0.046 | 6.16274431E-09 | 0.010 | 0.040 | 0.104 | PurS |
| 122 | Y | 0.919 | 0.000 | 5.28506838E-08 | 0.000 | 0.000 | 0.037 | PurS |
| 123 | R | 1.409 | 0.107 | 1.45440341E-08 | 0.030 | 0.076 | 0.155 | PurS |
| 124 | I | 1.610 | 0.211 | 1.30087373E-06 | 0.069 | 0.131 | 0.223 | PurS |
| 125 | P | 0.682 | 0.000 | 4.10256495E-10 | 0.000 | 0.000 | 0.051 | PurS |
| 126 | A | 0.941 | 0.282 | 0.001349581115 | 0.166 | 0.300 | 0.494 | PurS |
| 127 | D | 1.918 | 0.033 | 2.10942375E-15 | 0.003 | 0.017 | 0.054 | PurS |
| 128 | V | 0.699 | 0.036 | 8.91317954E-07 | 0.009 | 0.052 | 0.163 | PurS |
| 129 | D | 1.425 | 0.068 | 7.75281173E-09 | 0.015 | 0.047 | 0.111 | PurS |
| 130 | P | 0.698 | 0.018 | 1.47305261E-08 | 0.001 | 0.026 | 0.116 | PurS |
| 131 | L | 1.334 | 0.387 | 0.000695115512 | 0.168 | 0.290 | 0.466 | PurS |
| 132 | T | 1.080 | 0.625 | 0.117145535784 | 0.379 | 0.579 | 0.844 | NE |
| 133 | I | 0.631 | 0.122 | 0.001298341979 | 0.088 | 0.194 | 0.365 | PurS |
| 134 | T | 0.732 | 0.033 | 1.26930377E-07 | 0.007 | 0.045 | 0.140 | PurS |
| 135 | S | 1.153 | 0.056 | 4.8742832E-10 | 0.012 | 0.049 | 0.127 | PurS |
| 136 | S | 1.293 | 0.071 | 5.18622367E-10 | 0.017 | 0.055 | 0.130 | PurS |
| 137 | L | 0.405 | 0.046 | 0.000728328722 | 0.018 | 0.113 | 0.348 | PurS |
| 138 | S | 0.828 | 0.000 | 8.00193245E-12 | 0.000 | 0.000 | 0.040 | PurS |
| 139 | S | 0.591 | 0.095 | 0.00023368252 | 0.057 | 0.160 | 0.349 | PurS |
| 140 | D | 0.623 | 0.000 | 1.54682623E-08 | 0.000 | 0.000 | 0.049 | PurS |
| 141 | G | 1.884 | 0.017 | 7.77156117E-16 | 0.001 | 0.009 | 0.040 | PurS |
| 142 | V | 0.356 | 0.055 | 0.004948715863 | 0.038 | 0.155 | 0.404 | PurS |
| 143 | L | 0.435 | 0.000 | 2.05734779E-06 | 0.000 | 0.000 | 0.098 | PurS |
| 144 | T | 0.830 | 0.050 | 4.48851172E-08 | 0.015 | 0.060 | 0.156 | PurS |
| 145 | V | 1.003 | 0.089 | 1.36953126E-06 | 0.031 | 0.088 | 0.192 | PurS |
| 146 | N | 1.368 | 0.279 | 3.16769748E-05 | 0.118 | 0.204 | 0.330 | PurS |
| 147 | G | 0.355 | 0.093 | 0.013071514603 | 0.094 | 0.263 | 0.570 | PurS |

| | | | | | | | | |
|---|---|---|---|---|---|---|---|---|
| 148 | P | 0.749 | 0.037 | 1.08681758E-07 | 0.008 | 0.049 | 0.153 | PurS |
| 149 | R | 1.105 | 0.000 | 1.969791E-11 | 0.000 | 0.000 | 0.027 | PurS |
| 150 | K | 1.052 | 0.216 | 0.000560987549 | 0.112 | 0.205 | 0.342 | PurS |
| 151 | Q | 0.694 | 0.663 | 0.915204199804 | 0.601 | 0.956 | 1.458 | NE |
| 152 | V | 1.121 | 0.404 | 0.011080867899 | 0.220 | 0.361 | 0.558 | PurS |
| 153 | S | 2.044 | 0.371 | 2.24458155E-06 | 0.112 | 0.182 | 0.280 | PurS |
| 154 | G | 0.794 | 0.267 | 0.011134113858 | 0.182 | 0.336 | 0.565 | PurS |
| 155 | P | 1.663 | 0.114 | 1.03344111E-10 | 0.027 | 0.069 | 0.140 | PurS |
| 156 | E | 1.055 | 0.000 | 8.01897104E-09 | 0.000 | 0.000 | 0.030 | PurS |
| 157 | R | 0.736 | 0.036 | 7.39380138E-07 | 0.008 | 0.049 | 0.153 | PurS |
| 158 | T | 0.509 | 0.495 | 0.949029544393 | 0.630 | 0.972 | 1.448 | NE |
| 159 | I | 0.717 | 0.015 | 3.18556695E-08 | 0.001 | 0.021 | 0.093 | PurS |
| 160 | P | 0.840 | 0.093 | 8.07481187E-06 | 0.040 | 0.111 | 0.241 | PurS |
| 161 | I | 0.550 | 0.000 | 2.30990656E-07 | 0.000 | 0.000 | 0.052 | PurS |
| 162 | T | 1.143 | 0.152 | 1.29645819E-06 | 0.063 | 0.133 | 0.243 | PurS |
| 163 | R | 1.244 | 0.074 | 7.83047294E-10 | 0.018 | 0.060 | 0.139 | PurS |
| 164 | E | 1.000 | 0.151 | 0.000122980009 | 0.072 | 0.151 | 0.275 | PurS |
| 165 | E | 0.972 | 0.188 | 0.0039756852 | 0.099 | 0.193 | 0.336 | PurS |
| 166 | K | 3.021 | 0.016 | 2.420286E-14 | 0.000 | 0.005 | 0.024 | PurS |
| 167 | P | 0.594 | 0.143 | 0.001538574266 | 0.102 | 0.240 | 0.470 | PurS |
| 168 | A | 0.912 | 0.105 | 5.77005155E-06 | 0.041 | 0.116 | 0.253 | PurS |
| 169 | V | 0.835 | 0.305 | 0.021003660038 | 0.201 | 0.366 | 0.609 | PurS |
| 170 | T | 1.031 | 0.384 | 0.006385399236 | 0.214 | 0.372 | 0.605 | PurS |
| 171 | A | 0.626 | 0.153 | 0.002736336979 | 0.104 | 0.245 | 0.482 | PurS |
| 172 | A | 0.627 | 0.423 | 0.324324818546 | 0.406 | 0.675 | 1.061 | NE |
| 173 | P | 1.588 | 0.234 | 1.42922347E-05 | 0.076 | 0.147 | 0.256 | PurS |
| 174 | K | 0.956 | 0.117 | 0.000342305992 | 0.053 | 0.123 | 0.239 | PurS |
| 175 | K | 1.353 | 0.000 | 4.24480007E-10 | 0.000 | 0.000 | 0.022 | PurS |

**HspB8**

| site | amino acid | alpha | beta | p-value | dN/dS LB | dN/dS MLE (omega) | dN/dS UB | selection |
|---|---|---|---|---|---|---|---|---|
| 1 | M | 0.000 | 0.000 | n/a | n/a | n/a | n/a | ND † |
| 2 | A | 0.585 | 0.034 | 3.57680317E-07 | 0.010 | 0.059 | 0.182 | PurS |
| 3 | D | 3.454 | 0.043 | 0 | 0.003 | 0.012 | 0.032 | PurS |
| 4 | G | 0.500 | 0.094 | 0.000810747763 | 0.075 | 0.188 | 0.383 | PurS |
| 5 | Q | 0.831 | 0.031 | 6.27089586E-06 | 0.006 | 0.037 | 0.116 | PurS |
| 6 | M | 2.524 | 0.246 | 0.001440952995 | 0.053 | 0.097 | 0.162 | PurS |
| 7 | P | 0.858 | 0.000 | 1.92337867E-08 | 0.000 | 0.000 | 0.055 | PurS |
| 8 | F | 1.607 | 0.000 | 1.07503562E-10 | 0.000 | 0.000 | 0.022 | PurS |

| | | | | | | | | |
|---|---|---|---|---|---|---|---|---|
| 9 | S | 0.867 | 0.229 | 0.00559005442 | 0.126 | 0.264 | 0.483 | PurS |
| 10 | C | 1.353 | 0.000 | 1.04499537E-06 | 0.000 | 0.000 | 0.029 | PurS |
| 11 | H | 1.135 | 0.157 | 1.12514134E-05 | 0.071 | 0.138 | 0.239 | PurS |
| 12 | Y | 1.550 | 0.125 | 1.14596988E-06 | 0.037 | 0.081 | 0.152 | PurS |
| 13 | P | 1.545 | 0.112 | 7.43183293E-12 | 0.031 | 0.072 | 0.141 | PurS |
| 14 | S | 0.793 | 0.192 | 0.000841951604 | 0.128 | 0.242 | 0.410 | PurS |
| 15 | R | 0.864 | 0.030 | 1.37158718E-09 | 0.006 | 0.035 | 0.109 | PurS |
| 16 | L | 0.617 | 0.146 | 0.007011233344 | 0.107 | 0.237 | 0.445 | PurS |
| 17 | R | 0.623 | 0.000 | 2.34679787E-09 | 0.000 | 0.000 | 0.045 | PurS |
| 18 | R | 1.818 | 0.000 | 0 | 0.000 | 0.000 | 0.017 | PurS |
| 19 | D | 0.639 | 0.014 | 1.0312471E-06 | 0.001 | 0.021 | 0.095 | PurS |
| 20 | P | 1.081 | 0.016 | 8.5997875E-13 | 0.001 | 0.015 | 0.066 | PurS |
| 21 | F | 0.988 | 0.024 | 5.43242618E-09 | 0.004 | 0.025 | 0.077 | PurS |
| 22 | R | 1.042 | 0.085 | 3.28579641E-09 | 0.029 | 0.082 | 0.178 | PurS |
| 23 | D | 1.060 | 0.074 | 1.0893039E-06 | 0.025 | 0.070 | 0.151 | PurS |
| 24 | S | 1.468 | 0.180 | 1.71675839E-08 | 0.061 | 0.122 | 0.217 | PurS |
| 25 | P | 0.795 | 0.244 | 0.003620292748 | 0.171 | 0.307 | 0.505 | PurS |
| 26 | L | 1.158 | 0.053 | 1.21014543E-09 | 0.011 | 0.046 | 0.119 | PurS |
| 27 | S | 1.156 | 0.250 | 3.54757927E-05 | 0.121 | 0.216 | 0.355 | PurS |
| 28 | S | 1.096 | 0.031 | 1.31672451E-12 | 0.005 | 0.029 | 0.089 | PurS |
| 29 | R | 0.983 | 0.016 | 9.20019616E-12 | 0.001 | 0.017 | 0.074 | PurS |
| 30 | L | 0.516 | 0.031 | 4.04511216E-06 | 0.010 | 0.060 | 0.185 | PurS |
| 31 | L | 0.193 | 0.031 | 0.020919542058 | 0.027 | 0.161 | 0.501 | PurS |
| 32 | D | 3.780 | 0.087 | 7.77156117E-16 | 0.009 | 0.023 | 0.047 | PurS |
| 33 | D | 0.367 | 0.070 | 0.003839626495 | 0.068 | 0.190 | 0.411 | PurS |
| 34 | G | 0.875 | 0.124 | 9.70322598E-05 | 0.064 | 0.141 | 0.265 | PurS |
| 35 | F | 0.675 | 0.000 | 2.49353205E-10 | 0.000 | 0.000 | 0.034 | PurS |
| 36 | G | 0.492 | 0.068 | 0.000299615314 | 0.042 | 0.138 | 0.321 | PurS |
| 37 | M | 4.038 | 0.077 | 1.40883273E-05 | 0.008 | 0.019 | 0.037 | PurS |
| 38 | D | 1.203 | 0.147 | 2.98474177E-06 | 0.056 | 0.122 | 0.228 | PurS |
| 39 | P | 0.647 | 0.085 | 1.89730999E-05 | 0.047 | 0.131 | 0.285 | PurS |
| 40 | F | 1.353 | 0.000 | 2.29449792E-12 | 0.000 | 0.000 | 0.017 | PurS |
| 41 | P | 1.618 | 0.177 | 4.30638436E-09 | 0.055 | 0.110 | 0.194 | PurS |
| 42 | D | 2.351 | 0.161 | 4.80163176E-09 | 0.034 | 0.068 | 0.122 | PurS |
| 43 | D | 1.356 | 0.070 | 2.08701704E-08 | 0.018 | 0.051 | 0.112 | PurS |
| 44 | L | 1.019 | 0.037 | 5.10957165E-10 | 0.006 | 0.036 | 0.114 | PurS |
| 45 | T | 1.732 | 0.125 | 3.78075349E-12 | 0.033 | 0.072 | 0.137 | PurS |
| 46 | A | 0.838 | 0.115 | 4.52610857E-05 | 0.062 | 0.137 | 0.256 | PurS |
| 47 | S | 0.927 | 0.276 | 0.00304654862 | 0.178 | 0.298 | 0.465 | PurS |
| 48 | W | 0.000 | 0.016 | n/a | n/a | n/a | n/a | ND † |

| | | | | | | | | |
|---|---|---|---|---|---|---|---|---|
| 49 | P | 1.878 | 0.081 | 8.77076189E-15 | 0.015 | 0.043 | 0.094 | PurS |
| 50 | D | 1.126 | 0.118 | 1.2153606E-05 | 0.048 | 0.105 | 0.197 | PurS |
| 51 | W | 0.000 | 0.000 | n/a | n/a | n/a | n/a | ND † |
| 52 | A | 0.631 | 0.069 | 4.39155462E-06 | 0.034 | 0.110 | 0.258 | PurS |
| 53 | L | 0.686 | 0.037 | 1.14822131E-07 | 0.009 | 0.053 | 0.166 | PurS |
| 54 | P | 0.798 | 0.033 | 7.67514974E-09 | 0.007 | 0.042 | 0.129 | PurS |
| 55 | R | 1.274 | 0.033 | 6.650236E-14 | 0.004 | 0.026 | 0.081 | PurS |
| 56 | L | 0.794 | 0.080 | 9.56313077E-07 | 0.036 | 0.101 | 0.218 | PurS |
| 57 | S | 0.369 | 0.263 | 0.460210025232 | 0.414 | 0.714 | 1.142 | NE |
| 58 | S | 2.117 | 0.208 | 1.20136123E-11 | 0.053 | 0.098 | 0.169 | PurS |
| 59 | A | 1.174 | 0.263 | 3.05258909E-05 | 0.128 | 0.224 | 0.363 | PurS |
| 60 | W | 2.507 | 0.030 | 1.19000213E-07 | 0.002 | 0.012 | 0.038 | PurS |
| 61 | P | 2.258 | 0.196 | 1.11232135E-11 | 0.046 | 0.087 | 0.148 | PurS |
| 62 | G | 1.383 | 0.101 | 4.93671215E-10 | 0.029 | 0.073 | 0.149 | PurS |
| 63 | T | 0.644 | 0.376 | 0.130098848587 | 0.368 | 0.585 | 0.884 | NE |
| 64 | L | 0.521 | 0.020 | 1.42444897E-06 | 0.002 | 0.038 | 0.170 | PurS |
| 65 | R | 1.151 | 0.044 | 1.39295242E-11 | 0.009 | 0.038 | 0.099 | PurS |
| 66 | S | 1.786 | 0.291 | 1.09184067E-08 | 0.094 | 0.163 | 0.261 | PurS |
| 67 | G | 1.013 | 0.000 | 3.2041036E-13 | 0.000 | 0.000 | 0.031 | PurS |
| 68 | M | 1.442 | 0.127 | 5.23945232E-05 | 0.044 | 0.088 | 0.155 | PurS |
| 69 | V | 0.760 | 0.292 | 0.0153544165 | 0.223 | 0.384 | 0.615 | PurS |
| 70 | P | 0.874 | 0.224 | 0.001567104368 | 0.126 | 0.257 | 0.463 | PurS |
| 71 | R | 1.331 | 0.211 | 2.29399204E-07 | 0.083 | 0.158 | 0.269 | PurS |
| 72 | G | 0.808 | 0.326 | 0.029599721699 | 0.237 | 0.403 | 0.640 | PurS |
| 73 | P | 0.899 | 0.065 | 4.63554456E-08 | 0.022 | 0.073 | 0.170 | PurS |
| 74 | T | 1.759 | 0.415 | 4.64454525E-06 | 0.150 | 0.236 | 0.354 | PurS |
| 75 | A | 1.233 | 0.442 | 0.00277675509 | 0.225 | 0.358 | 0.544 | PurS |
| 76 | T | 1.222 | 0.475 | 0.004136516545 | 0.257 | 0.389 | 0.568 | PurS |
| 77 | A | 0.646 | 0.550 | 0.680017714408 | 0.566 | 0.852 | 1.236 | NE |
| 78 | R | 1.940 | 0.127 | 5.95749006E-11 | 0.030 | 0.065 | 0.121 | PurS |
| 79 | F | 0.853 | 0.128 | 0.000202940246 | 0.072 | 0.150 | 0.273 | PurS |
| 80 | G | 1.144 | 0.221 | 7.29295223E-05 | 0.104 | 0.193 | 0.323 | PurS |
| 81 | V | 1.526 | 0.156 | 1.19428122E-08 | 0.049 | 0.102 | 0.186 | PurS |
| 82 | P | 1.006 | 0.246 | 0.000162421667 | 0.136 | 0.244 | 0.401 | PurS |
| 83 | A | 3.170 | 0.297 | 4.29067892E-12 | 0.052 | 0.094 | 0.155 | PurS |
| 84 | E | 1.194 | 0.166 | 1.64934232E-05 | 0.069 | 0.139 | 0.246 | PurS |
| 85 | G | 0.806 | 0.443 | 0.118390402424 | 0.347 | 0.550 | 0.829 | NE |
| 86 | R | 0.985 | 0.236 | 0.000393611086 | 0.133 | 0.239 | 0.397 | PurS |
| 87 | T | 1.609 | 0.297 | 0.001138846569 | 0.112 | 0.184 | 0.285 | PurS |
| 88 | P | 1.054 | 0.356 | 0.001956921931 | 0.207 | 0.337 | 0.520 | PurS |

| | | | | | | | | |
|---|---|---|---|---|---|---|---|---|
| 89 | P | 0.963 | 0.404 | 0.009959429758 | 0.260 | 0.420 | 0.643 | PurS |
| 90 | P | 1.088 | 0.342 | 0.002434482676 | 0.188 | 0.315 | 0.493 | PurS |
| 91 | F | 1.887 | 0.370 | 7.3801799E-05 | 0.126 | 0.196 | 0.290 | PurS |
| 92 | P | 1.428 | 0.349 | 2.31287772E-05 | 0.147 | 0.244 | 0.378 | PurS |
| 93 | G | 1.023 | 0.068 | 3.64922916E-08 | 0.020 | 0.066 | 0.155 | PurS |
| 94 | E | 0.962 | 0.015 | 1.82336956E-07 | 0.001 | 0.015 | 0.068 | PurS |
| 95 | P | 0.805 | 0.016 | 3.11954762E-09 | 0.001 | 0.020 | 0.088 | PurS |
| 96 | W | 0.000 | 0.000 | n/a | n/a | n/a | n/a | ND † |
| 97 | K | 0.725 | 0.000 | 2.27151742E-10 | 0.000 | 0.000 | 0.033 | PurS |
| 98 | V | 1.013 | 0.000 | 2.41362486E-12 | 0.000 | 0.000 | 0.031 | PurS |
| 99 | C | 1.616 | 0.000 | 0 | 0.000 | 0.000 | 0.015 | PurS |
| 100 | V | 0.526 | 0.000 | 5.78862396E-08 | 0.000 | 0.000 | 0.059 | PurS |
| 101 | N | 2.633 | 0.000 | 0 | 0.000 | 0.000 | 0.009 | PurS |
| 102 | V | 0.444 | 0.000 | 6.96555825E-07 | 0.000 | 0.000 | 0.070 | PurS |
| 103 | H | 0.533 | 0.084 | 0.00688350862 | 0.062 | 0.157 | 0.320 | PurS |
| 104 | S | 0.628 | 0.035 | 7.03913067E-05 | 0.014 | 0.055 | 0.144 | PurS |
| 105 | F | 0.984 | 0.012 | 3.60270952E-08 | 0.001 | 0.012 | 0.054 | PurS |
| 106 | K | 1.037 | 0.136 | 5.0881533E-06 | 0.065 | 0.131 | 0.232 | PurS |
| 107 | P | 1.653 | 0.053 | 5.55111512E-16 | 0.008 | 0.032 | 0.082 | PurS |
| 108 | E | 0.533 | 0.015 | 8.79977072E-05 | 0.002 | 0.028 | 0.123 | PurS |
| 109 | E | 1.662 | 0.000 | 7.561729E-13 | 0.000 | 0.000 | 0.017 | PurS |
| 110 | L | 0.490 | 0.019 | 2.98943635E-06 | 0.002 | 0.039 | 0.176 | PurS |
| 111 | M | 2.236 | 0.141 | 1.9480318E-08 | 0.033 | 0.063 | 0.109 | PurS |
| 112 | V | 1.290 | 0.049 | 3.84473564E-11 | 0.009 | 0.038 | 0.099 | PurS |
| 113 | K | 0.698 | 0.012 | 3.16344611E-08 | 0.001 | 0.018 | 0.078 | PurS |
| 114 | T | 0.426 | 0.000 | 3.18979568E-07 | 0.000 | 0.000 | 0.063 | PurS |
| 115 | K | 0.699 | 0.052 | 8.28729232E-06 | 0.023 | 0.074 | 0.173 | PurS |
| 116 | D | 1.693 | 0.000 | 0 | 0.000 | 0.000 | 0.016 | PurS |
| 117 | G | 1.123 | 0.017 | 3.3029135E-13 | 0.001 | 0.015 | 0.066 | PurS |
| 118 | Y | 1.901 | 0.041 | 2.6711966E-13 | 0.005 | 0.021 | 0.056 | PurS |
| 119 | V | 0.412 | 0.000 | 3.37959582E-07 | 0.000 | 0.000 | 0.075 | PurS |
| 120 | E | 0.829 | 0.014 | 8.32049011E-08 | 0.001 | 0.017 | 0.077 | PurS |
| 121 | V | 0.703 | 0.016 | 2.45583039E-08 | 0.001 | 0.023 | 0.101 | PurS |
| 122 | S | 0.456 | 0.000 | 8.62582821E-08 | 0.000 | 0.000 | 0.077 | PurS |
| 123 | G | 0.819 | 0.000 | 1.08295595E-11 | 0.000 | 0.000 | 0.039 | PurS |
| 124 | K | 0.479 | 0.074 | 0.000547724074 | 0.061 | 0.155 | 0.314 | PurS |
| 125 | H | 4.476 | 0.000 | 0 | 0.000 | 0.000 | 0.006 | PurS |
| 126 | E | 0.425 | 0.000 | 6.43873085E-07 | 0.000 | 0.000 | 0.063 | PurS |
| 127 | E | 1.353 | 0.000 | 3.16568993E-12 | 0.000 | 0.000 | 0.021 | PurS |
| 128 | K | 1.366 | 0.040 | 9.03746522E-10 | 0.007 | 0.029 | 0.076 | PurS |

| | | | | | | | | |
|---|---|---|---|---|---|---|---|---|
| 129 | Q | 1.353 | 0.000 | 2.79312129E-11 | 0.000 | 0.000 | 0.022 | PurS |
| 130 | Q | 1.150 | 0.270 | 0.000307338785 | 0.139 | 0.235 | 0.370 | PurS |
| 131 | E | 0.439 | 0.028 | 6.64141881E-05 | 0.011 | 0.064 | 0.199 | PurS |
| 132 | G | 0.993 | 0.000 | 2.708944E-14 | 0.000 | 0.000 | 0.033 | PurS |
| 133 | G | 1.518 | 0.017 | 1.565414E-14 | 0.001 | 0.011 | 0.049 | PurS |
| 134 | I | 1.782 | 0.012 | 0 | 0.000 | 0.007 | 0.029 | PurS |
| 135 | V | 0.641 | 0.016 | 2.03130116E-08 | 0.001 | 0.025 | 0.111 | PurS |
| 136 | S | 0.681 | 0.031 | 7.37678355E-08 | 0.008 | 0.045 | 0.141 | PurS |
| 137 | K | 0.648 | 0.000 | 8.59509591E-08 | 0.000 | 0.000 | 0.037 | PurS |
| 138 | N | 0.743 | 0.000 | 5.13548504E-09 | 0.000 | 0.000 | 0.030 | PurS |
| 139 | F | 0.208 | 0.000 | 0.002759623822 | 0.000 | 0.000 | 0.111 | PurS |
| 140 | T | 0.533 | 0.000 | 1.29430278E-09 | 0.000 | 0.000 | 0.051 | PurS |
| 141 | K | 0.326 | 0.000 | 0.000201152759 | 0.000 | 0.000 | 0.075 | PurS |
| 142 | K | 0.535 | 0.000 | 2.53054003E-08 | 0.000 | 0.000 | 0.044 | PurS |
| 143 | I | 0.874 | 0.012 | 1.9701385E-10 | 0.001 | 0.014 | 0.059 | PurS |
| 144 | Q | 0.775 | 0.016 | 6.99126721E-06 | 0.001 | 0.021 | 0.094 | PurS |
| 145 | L | 0.676 | 0.060 | 1.01528513E-06 | 0.027 | 0.089 | 0.208 | PurS |
| 146 | P | 1.077 | 0.000 | 4.99600361E-15 | 0.000 | 0.000 | 0.028 | PurS |
| 147 | A | 1.046 | 0.505 | 0.042922570528 | 0.313 | 0.483 | 0.715 | PurS |
| 148 | E | 1.798 | 0.043 | 3.33211236E-12 | 0.006 | 0.024 | 0.063 | PurS |
| 149 | V | 0.258 | 0.000 | 8.76836051E-05 | 0.000 | 0.000 | 0.120 | PurS |
| 150 | D | 1.052 | 0.014 | 2.92816882E-11 | 0.001 | 0.013 | 0.058 | PurS |
| 151 | P | 0.769 | 0.032 | 3.41804884E-09 | 0.007 | 0.042 | 0.131 | PurS |
| 152 | V | 0.519 | 0.398 | 0.516926257925 | 0.477 | 0.767 | 1.170 | NE |
| 153 | T | 1.448 | 0.042 | 1.55431223E-15 | 0.007 | 0.029 | 0.076 | PurS |
| 154 | V | 1.963 | 0.000 | 0 | 0.000 | 0.000 | 0.016 | PurS |
| 155 | F | 1.353 | 0.000 | 8.8817842E-16 | 0.000 | 0.000 | 0.017 | PurS |
| 156 | A | 0.626 | 0.000 | 3.03259196E-09 | 0.000 | 0.000 | 0.052 | PurS |
| 157 | S | 1.353 | 0.000 | 0 | 0.000 | 0.000 | 0.023 | PurS |
| 158 | L | 0.990 | 0.018 | 9.43423117E-12 | 0.001 | 0.018 | 0.079 | PurS |
| 159 | S | 1.353 | 0.000 | 2.88657986E-15 | 0.000 | 0.000 | 0.023 | PurS |
| 160 | P | 0.818 | 0.000 | 1.19182442E-12 | 0.000 | 0.000 | 0.037 | PurS |
| 161 | E | 1.599 | 0.000 | 5.484502E-14 | 0.000 | 0.000 | 0.017 | PurS |
| 162 | G | 1.353 | 0.000 | 0 | 0.000 | 0.000 | 0.023 | PurS |
| 163 | L | 0.426 | 0.018 | 7.58169624E-06 | 0.002 | 0.042 | 0.185 | PurS |
| 164 | L | 0.696 | 0.000 | 5.03075359E-10 | 0.000 | 0.000 | 0.049 | PurS |
| 165 | I | 0.505 | 0.000 | 1.987019E-07 | 0.000 | 0.000 | 0.046 | PurS |
| 166 | I | 0.852 | 0.000 | 4.74873474E-11 | 0.000 | 0.000 | 0.027 | PurS |
| 167 | E | 0.707 | 0.000 | 1.58597413E-09 | 0.000 | 0.000 | 0.038 | PurS |
| 168 | A | 1.191 | 0.034 | 5.1303406E-13 | 0.005 | 0.029 | 0.089 | PurS |

| | | | | | | | | |
|---|---|---|---|---|---|---|---|---|
| 169 | P | 1.030 | 0.017 | 5.83666449E-12 | 0.001 | 0.016 | 0.071 | PurS |
| 170 | Q | 1.662 | 0.067 | 1.00296615E-07 | 0.013 | 0.041 | 0.095 | PurS |
| 171 | V | 2.398 | 0.170 | 3.68594E-14 | 0.037 | 0.071 | 0.121 | PurS |
| 172 | P | 0.907 | 0.000 | 3.7592152E-13 | 0.000 | 0.000 | 0.034 | PurS |
| 173 | P | 1.353 | 0.000 | 4.4408921E-16 | 0.000 | 0.000 | 0.023 | PurS |
| 174 | Y | 1.631 | 0.031 | 4.73344697E-11 | 0.003 | 0.019 | 0.058 | PurS |
| 175 | S | 0.756 | 0.245 | 0.007861271521 | 0.181 | 0.324 | 0.531 | PurS |
| 176 | T | 0.937 | 0.266 | 0.000614493669 | 0.161 | 0.284 | 0.458 | PurS |
| 177 | F | 0.913 | 0.187 | 0.000224874869 | 0.112 | 0.204 | 0.339 | PurS |
| 178 | G | 0.412 | 0.187 | 0.084630863697 | 0.237 | 0.455 | 0.780 | PurS |
| 179 | E | 1.778 | 0.357 | 0.000613450486 | 0.127 | 0.201 | 0.301 | PurS |
| 180 | S | 0.608 | 0.383 | 0.400169223879 | 0.410 | 0.630 | 0.924 | NE |
| 181 | S | 1.041 | 0.586 | 0.13714742828 | 0.393 | 0.562 | 0.785 | NE |
| 182 | F | 1.565 | 0.368 | 0.000440335008 | 0.148 | 0.235 | 0.352 | PurS |
| 183 | N | 1.019 | 0.415 | 0.026661066853 | 0.263 | 0.407 | 0.600 | PurS |
| 184 | N | 7.278 | 0.417 | 3.21964677E-15 | 0.038 | 0.057 | 0.083 | PurS |
| 185 | E | 0.710 | 0.154 | 0.006380884445 | 0.108 | 0.217 | 0.384 | PurS |
| 186 | L | 0.645 | 0.492 | 0.484256073343 | 0.505 | 0.763 | 1.106 | NE |
| 187 | P | 1.452 | 0.353 | 0.000217011784 | 0.146 | 0.243 | 0.379 | PurS |
| 188 | Q | 1.889 | 0.353 | 1.49643124E-05 | 0.115 | 0.187 | 0.287 | PurS |
| 189 | D | 0.821 | 0.214 | 0.015173111672 | 0.146 | 0.260 | 0.424 | PurS |
| 190 | S | 0.369 | 0.414 | 0.805903280041 | 0.739 | 1.121 | 1.632 | NE |
| 191 | Q | 1.921 | 0.200 | 1.63299297E-07 | 0.055 | 0.104 | 0.177 | PurS |
| 192 | E | 0.511 | 0.043 | 0.000113392208 | 0.021 | 0.084 | 0.219 | PurS |
| 193 | V | 0.747 | 0.185 | 0.00204307105 | 0.123 | 0.247 | 0.440 | PurS |
| 194 | T | 0.869 | 0.231 | 0.000493054586 | 0.151 | 0.266 | 0.429 | PurS |
| 195 | C | 0.945 | 0.149 | 1.15963714E-05 | 0.078 | 0.157 | 0.278 | PurS |
| 196 | T | 0.485 | 0.205 | 0.055919536973 | 0.224 | 0.423 | 0.719 | PurS |
