# Supplementary material for: Human HspB1, HspB3, HspB5 and HspB8: Shaping these disease factors during vertebrate evolution
Source: Cell Stress Chaperones. 2022 Jun 9;27(4):309–23. doi: 10.1007/s12192-022-01268-y (PMC9346038; doi:10.1007/s12192-022-01268-y)
Supplement: Supplementary file 5 — (PDF 493 KB) [file 12192_2022_1268_MOESM5_ESM.pdf]

Online Supplemental Materials

Table S4. Aggregate $\overline{\omega}$-values as returned by the FitMG94 algorithm of full-length sequences and of selected sequence partitions of human HspB1, HspB3, HspB5 and HspB8

sHSP	full-length sequence/ sequence partition	region	codons excluded from calculation	$\overline{\omega}$ (aggregate omega)	confidence interval (lower bound)	confidence interval (upper bound)
**HspB1**	M1 - K205	full-length	M1, F8, W16	$\overline{\omega}_{FL}$ = 0.127	0.122	0.133
	M1 - P57	N-terminal region	M1, F8, W16	$\overline{\omega}_{NTR}$ = 0.104	0.095	0.113
	L58 - S78	central region		$\overline{\omega}_{CeR}$ = 0.232	0.212	0.254
	R79 - P168	α-crystallin domain		$\overline{\omega}_{\alpha CD}$ = 0.069	0.063	0.075
	M169 - K205	C-terminal extension		$\overline{\omega}_{CTE}$ = 0.190	0.173	0.207
	M1 - G122	exon 1	M1, F8, W16	$\overline{\omega}_{Ex1}$ = 0.118	0.112	0.125
	K123 - Y143	exon 2		$\overline{\omega}_{Ex2}$ = 0.032	0.024	0.041
	T144 - K205	exon 3		$\overline{\omega}_{Ex3}$ = 0.146	0.135	0.158
**HspB3**	M1 - K150	full-length	M1, W93, M104	$\overline{\omega}_{FL}$ = 0.231	0.220	0.242
	M1 - P40	N-terminal region	M1	$\overline{\omega}_{NTR}$ = 0.150	0.134	0.168
	T41 - H70	central region		$\overline{\omega}_{CeR}$ = 0.610	0.567	0.656
	F71 - L139	α-crystallin domain	W93, M104	$\overline{\omega}_{\alpha CD}$ = 0.086	0.076	0.097
	V140 - K150	C-terminal extension		$\overline{\omega}_{CTE}$ = 0.416	0.350	0.491
**HspB5**	M1 - K175	full-length	M1, W60, Q108	$\overline{\omega}_{FL}$ = 0.120	0.113	0.127
	M1 - P51	N-terminal region	M1	$\overline{\omega}_{NTR}$ = 0.106	0.096	0.117
	P52 - T63	central region	W60	$\overline{\omega}_{CeR}$ = 0.223	0.183	0.269
	G64 - R149	α-crystallin domain	Q108	$\overline{\omega}_{\alpha CD}$ = 0.084	0.076	0.093
	K150 - K175	C-terminal extension		$\overline{\omega}_{CTE}$ = 0.210	0.183	0.238
	M1 - E67	exon 1	M1, W60	$\overline{\omega}_{Ex1}$ = 0.126	0.116	0.137
	M68 - R107	exon 2		$\overline{\omega}_{Ex2}$ = 0.086	0.074	0.099
	Q108 - K175	exon 3	Q108	$\overline{\omega}_{Ex3}$ = 0.134	0.122	0.147
**HspB8**	M1 - T196	full-length	M1, W48, W51, W96	$\overline{\omega}_{FL}$ = 0.109	0.103	0.115
	M1 - M68	N-terminal region	M1, W48, W51	$\overline{\omega}_{NTR}$ = 0.092	0.083	0.101
	V69 - G93	central region		$\overline{\omega}_{CeR}$ = 0.194	0.174	0.215
	E94 - Y174	α-crystallin domain	W96	$\overline{\omega}_{\alpha CD}$ = 0.037	0.032	0.043
	S175 - T196	C-terminal extension		$\overline{\omega}_{CTE}$ = 0.272	0.244	0.303
	M1 - G123	exon 1	M1, W48, W51, W96	$\overline{\omega}_{Ex1}$ = 0.106	0.099	0.113
	K124 - I143	exon 2		$\overline{\omega}_{Ex2}$ = 0.035	0.024	0.050
	Q144 - T196	exon 3		$\overline{\omega}_{Ex3}$ = 0.145	0.132	0.158
