# Supplementary material for: Human HspB1, HspB3, HspB5 and HspB8: Shaping these disease factors during vertebrate evolution
Source: Cell Stress Chaperones. 2022 Jun 9;27(4):309–23. doi: 10.1007/s12192-022-01268-y (PMC9346038; doi:10.1007/s12192-022-01268-y)
Supplement: Supplementary file 6 — (PDF 95 KB) [file 12192_2022_1268_MOESM6_ESM.pdf]

Online Supplemental Materials

Table S5. Examples for reported molecular and cellular implications of sHSP missense mutations with potential toxic gain-of-function mechanism. All listed mutations are associated with dominant or semi-dominant neuromuscular disease phenotypes. The molecular and cellular consequences fall into four categories, all known dominant gain-of-function mechanisms (Wilkie et al. 1994[a]): (1) dominant-negative effects through altered protein-protein interactions (impaired quaternary structure), (2) accumulation of toxic products (e.g. formation of protein aggregatess and their highly toxic amyloid precursors), (3) impaired cell architecture (e.g. through modified cytoskeletal components), and (4) increased enzymatic (downstream) activities.

sHSP	Mutation	Observed Molecular and Cellular Gain-of-Function Mechanisms	References[a]
HspB1	p.P7S	hyperphosphorylation of neurofilaments	Echaniz-Laguna et al. 2017
	p.R127W	increased chaperone activity; increased fraction of the protein residing in the monomeric state; enhanced binding to tubulin	Almeida-Souza et al. 2010; 2011
		abnormal neurofilament staining pattern and aggregation	Zhang et al. 2011
		increased propensity for large oligomeric complex formation at high concentrations; increased propensity to dissociate at low concentrations	Weeks et al. 2018
		impaired formation of SQSTM1/p62 bodies in autophagy	Haidar et al. 2019
	p.Q128R	hyperphosphorylation of neurofilaments	Echaniz-Laguna et al. 2017
	p.S135F	impaired neurofilament assembly; formation of protein aggregates	Evgrafov et al. 2004
		increased interaction with HspB8	Fontaine et al. 2006
		disruption of neurofilament network and aggregation of neurofilament light chain, resulting in progressive degeneration of motor neurons	Zhai et al. 2007
		increased chaperone activity; increased fraction of the protein residing in the monomeric state; enhanced binding to tubulin; abnormal stabilization of microtubules	Almeida-Souza et al. 2010; 2011
		decreased level of acetylated α-tubulin possibly resulting from increased activity of histone deacetylase in sciatic nerve; impaired axonal transport	d'Ydewalle et al. 2011
		hyperphosphorylation of cyclin-dependent protein kinase Cdk5 resulting in hyperphosphorylation of neurofilaments, affecting their binding to the motor protein kinesin and thereby the anterograde transport	Holmgren et al. 2013
		increased propensity for large oligomeric complex formation at high concentrations; increased propensity to dissociate at low concentrations	Weeks et al. 2018
		impaired formation of SQSTM1/p62 bodies in autophagy	Haidar et al. 2019
	p.R136W	increased chaperone activity; increased fraction of the protein residing in the monomeric state; enhanced binding to tubulin	Almeida-Souza et al. 2010; 2011
		increased propensity for large oligomeric complex formation at high concentrations, increased propensity to dissociate at low concentrations	Weeks et al. 2018
	p.P182L	formation of protein aggregates (sequestration of neurofilament middle chain subunit and p150 dynactin); disruption of neurofilament assembly and axonal transport	Ackerley et al. 2006
		decreased level of acetylated α-tubulin possibly resulting from increased activity of histone deacetylase in sciatic nerve; impaired axonal transport	d'Ydewalle et al. 2011
		hyperphosphorylation of cyclin-dependent protein kinase Cdk5 resulting in hyperphosphorylation of neurofilaments, affecting their binding to the motor protein kinesin and thereby the anterograde transport	Holmgren et al. 2013

		increased interaction with the RNA binding protein PCBP1, resulting in reduced translational repressive activity	Geuens et al. 2017
		impaired formation of SQSTM1/p62 bodies in autophagy	Haidar et al. 2019
	p.S187L	formation of protein aggregates	Echaniz-Laguna et al. 2017
HspB8	p.K141E	formation of protein aggregates	Irobi et al. 2004
		altered (largely increased) sHSP interactions; increased formation of protein aggregates	Fontaine et al. 2006
		increased interaction with the RNA-helicase Ddx20 (gemin 3)	Sun et al. 2010
		reduced interaction with BAG3	Shemetov and Gusev 2011
		impaired clearing of protein aggregates	Carra et al. 2010
	p.K141N	formation of protein aggregates	Irobi et al. 2004
		altered (largely increased) sHSP interactions; increased formation of protein aggregates	Fontaine et al. 2006
		increased interaction with the RNA-helicase Ddx20 (gemin 3)	Sun et al. 2010
		impaired clearing of protein aggregates	Carra et al. 2010
		formation of protein aggregates; reduced mitochondrial membrane potential	Irobi et al. 2012
		impact on the muscular cell architecture: Z-disk disorganization; granulofilamentous material accumulating along with mutant HspB8; desmin aggregation; impaired autophagy	Bouhy et al. 2018
	p.K141M	increased binding to BAG3	Echaniz-Laguna et al. 2017
HspB5	p.R120G	mutant HspB5-desmin aggregate formation	Vicart et al. 1998
		irregular quaternary structure of HspB5; defective chaperone function	Bova et al. 1999
		translocation of FBX4, a component of the ubiquitin-protein ligase system to the detergent-insoluble fraction and stimulation of ubiquitination	den Engelsman et al. 2003
		alterations in mitochondrial organization and architecture; impaired cytoskeletal network	Maloyan et al. 2005
		oxidative stress due to augmented expression or activity of glucose-6-phosphate dehydrogenase, glutathione reductase, and glutathione peroxidase; protein aggregation	Rajasekaran et al. 2007
		abnormal sHSP interactions; increased tendency for aggregate formation; increased degree of mutant HspB5 phosphorylation	Simon et al. 2007
		perinuclear formation of protein aggregates	Sanbe 2011
		promotes desmin filament aggregation	Elliott et al. 2013

[a]References:

Ackerley S, James PA, Kalli A, French S, Davies KE, Talbot K (2006) A mutation in the small heat-shock protein HSPB1 leading to distal hereditary motor neuronopathy disrupts neurofilament assembly and the axonal transport of specific cellular cargoes. Hum Mol Genet 15:347-354

Almeida-Souza L, Asselbergh B, d'Ydewalle C, Moonens K, Goethals S, de Winter V, Azmi A, Irobi J, Timmermans JP, Gevaert K, Remaut H, Van Den Bosch L, Timmerman V, Janssens S (2011) Small heat-shock protein HSPB1 mutants stabilize microtubules in Charcot-Marie-Tooth neuropathy. J Neurosci 31:15320-15328

Almeida-Souza L, Goethals S, Winter V De, Dierick I, Gallardo R, Durme J Van, Irobi J, Gettemans J, Rousseau F, Schymkowitz J, Timmerman V, Janssens S (2010) Increased monomerization of mutant HSPB1 leads to protein hyperactivity in Charcot-Marie-Tooth neuropathy. J Biol Chem 285:12778–12786

Bouhy D, Juneja M, Katona I, Holmgren A, Asselbergh B, De Winter V, Hochepied T, Goossens S, Haigh JJ, Libert C, Ceuterick-de Groote C, Irobi J, Weis J, Timmerman V (2018) A knock-in/knock-out mouse model of HSPB8-associated distal hereditary motor neuropathy and myopathy reveals toxic gain-of-function of mutant HspB8. Acta Neuropathol 135:131-148

Bova MP, Yaron O, Huang Q, Ding L, Haley DA, Stewart PL, Horwitz J. 1999. Mutation R120G in alphaB-crystallin, which is linked to a desmin-related myopathy, results in an irregular structure and defective chaperone-like function. Proc Natl Acad Sci U S A 96:6137-6142

Carra S, Boncoraglio A, Kanon B, Brunsting JF, Minoia M, Rana A, Vos MJ, Seidel K, Sibon OCM, Kampinga HH (2010) Identification of the Drosophila ortholog of HSPB8: implication of HSPB8 loss of function in protein folding diseases. J Biol Chem 285:37811–37822

den Engelsman J, Keijsers V, de Jong WW, Boelens WC (2003) The small heat-shock protein alpha B-crystallin promotes FBX4-dependent ubiquitination. J Biol Chem 278:4699-4704

d'Ydewalle C, Krishnan J, Chiheb DM, Van Damme P, Irobi J, Kozikowski AP, Vanden Berghe P, Timmerman V, Robberecht W, Van Den Bosch L (2011) HDAC6 inhibitors reverse axonal loss in a mousemodel ofmutant HSPB1-induced Charcot-Marie-Tooth disease. Nat Med 17:968–974

Echaniz-Laguna A, Geuens T, Petiot P, Péréon Y, Adriaenssens E, Haidar M, Capponi S, Maisonobe T, Fournier E, Dubourg O, Degos B, Salachas F, Lenglet T, Eymard B, Delmont E, Pouget J, Juntas Morales R, Goizet C, Latour P, Timmerman V, Stojkovic T (2017) Axonal Neuropathies due to Mutations in Small Heat Shock Proteins: Clinical, Genetic, and Functional Insights into Novel Mutations. Hum Mutat 38:556-568

Elliott JL, Der Perng M, Prescott AR, Jansen KA, Koenderink GH, Quinlan RA (2013) The specificity of the interaction between αB-crystallin and desmin filaments and its impact on filament aggregation and cell viability. Philos Trans R Soc Lond B Biol Sci 368 (1617)

Evgrafov OV, Mersiyanova I, Irobi J, Van Den Bosch L, Dierick I, Leung CL, Schagina O, Verpoorten N, Van Impe K, Fedotov V, Dadali E, Auer-Grumbach M, Windpassinger C, Wagner K, Mitrovic Z, Hilton-Jones D, Talbot K, Martin JJ, Vasserman N, Tverskaya S, Polyakov A, Liem RK, Gettemans J, Robberecht W, De Jonghe P, Timmerman V (2004) Mutant small heat-shock protein 27 causes axonal Charcot-Marie-Tooth disease and distal hereditary motor neuropathy. Nat Genet 36:602-606

Fontaine JM, Sun X, Hoppe AD, Simon S, Vicart P, Welsh MJ, Benndorf R (2006) Abnormal small heat shock protein interactions involving neuropathy-associated HSP22 (HSPB8) mutants. FASEB J 20:2168-2170

Geuens T, De Winter V, Rajan N, Achsel T, Mateiu L, Almeida-Souza L, Asselbergh B, Bouhy D, Auer-Grumbach M, Bagni C, Timmerman V (2017) Mutant HSPB1 causes loss of translational repression by binding to PCBP1, an RNA binding protein with a possible role in neurodegenerative disease. Acta Neuropathol Commun 5:5

Haidar M, Asselbergh B, Adriaenssens E, De Winter V, Timmermans JP, Auer-Grumbach M, Juneja M, Timmerman V (2019) Neuropathy-causing mutations in HSPB1 impair autophagy by disturbing the formation of SQSTM1/p62 bodies. Autophagy 15:1051-1068

Holmgren A, Bouhy D, De Winter V, Asselbergh B, Timmermans J-P, Irobi J, Timmerman V (2013) Charcot-Marie-Tooth causing HSPB1mutations increase Cdk5-mediated phosphorylation of neurofilaments. Acta Neuropathol 126:93–108

Irobi J, Van Impe K, Seeman P, Jordanova A, Dierick I, Verpoorten N, Michalik A, De Vriendt E, Jacobs A, Van Gerwen V, Vennekens K, Mazanec R, Tournev I, Hilton-Jones D, Talbot K, Kremensky I, Van Den Bosch L, Robberecht W, Van Vandekerckhove J, Van Broeckhoven C, Gettemans J, De Jonghe P, Timmerman V (2004) Hot-spot residue in small heat-shock protein 22 causes distal motor neuropathy. Nat Genet 36:597-601

Irobi J,Holmgren A, DeWinter V, Asselbergh B, Gettemans J, Adriaensen D, Ceuterickde Groote C, Van Coster R, De Jonghe P, Timmerman V (2012) Mutant HSPB8 causes protein aggregates and a reduced mitochondrial membrane potential in dermal fibroblasts from distalHereditaryMotor Neuropathy patients. Neuromuscular Disorders 22:699–711

Maloyan A, Sanbe A, Osinska H, Westfall M, Robinson D, Imahashi K, Murphy E, Robbins J (2005) Mitochondrial dysfunction and apoptosis underlie the pathogenic process in alphaB-crystallin desmin-related cardiomyopathy. Circulation 112:3451–3461

Rajasekaran NS, Connell P, Christians ES, Yan LJ, Taylor RP, Orosz A, Zhang XQ, Stevenson TJ, Peshock RM, Leopold JA, Barry WH, Loscalzo J, Odelberg SJ, Benjamin IJ (2007) Human alpha B-crystallin mutation causes oxido-reductive stress and protein aggregation cardiomyopathy in mice. Cell 130:427–439

Sanbe A (2011) Molecular mechanisms of alpha-crystallinopathy and its therapeutic strategy. Biol Pharm Bull 34 1653–1658

Shemetov AA, Gusev NB (2011) Biochemical characterization of small heat shock protein HspB8 (Hsp22)–Bag3 interaction. Arch Biochem Biophys 513:1–9

Simon S, Fontaine JM, Martin JL, Sun X, Hoppe AD, Welsh MJ, Benndorf R, Vicart P (2007) Myopathy-associated alphaB-crystallin mutants: abnormal phosphorylation, intracellular location, and interactions with other small heat shock proteins. J Biol Chem 282:34276-34287

Sun X, Fontaine JM, Hoppe AD, Carra S, DeGuzman C, Martin JL, Simon S, Vicart P, Welsh MJ, Landry J, Benndorf R (2010) Abnormal interaction of motor neuropathy-associated mutant HspB8 (Hsp22) forms with the RNA helicase Ddx20 (gemin3). Cell Stress Chaperones 15:567-582

Vicart P, Caron A, Guicheney P, Li Z, Prévost MC, Faure A, Chateau D, Chapon F, Tomé F, Dupret JM, Paulin D, Fardeau M (1998) A missense mutation in the alphaB-crystallin chaperone gene causes a desmin-related myopathy. Nat Genet 20:92-95

Weeks SD, Muranova LK, Heirbaut M, Beelen S, Strelkov SV, Gusev NB (2018) Characterization of human small heat shock protein HSPB1 alpha-crystallin domain localized mutants associated with hereditary motor neuron diseases. Sci Rep 8:688

Wilkie AO (1994) The molecular basis of genetic dominance. J Med Genet 31:89-98

Zhai J, Lin H, Julien JP, Schlaepfer WW (2007) Disruption of neurofilament network with aggregation of light neurofilament protein: a common pathway leading to motor neuron degeneration due to Charcot-Marie-Tooth disease-linked mutations in NFL and HSPB1. Hum Mol Genet 16:3103-3116

Zhang RX, Yang X, Zi XH, Li XB, Xia K, Liu T, Liu SM, Li L, Zhan YJ, Li L, Pan Q, Tang BS (2011) Cellular expression of (R127W) HSPB1 and its co-localization with neurofilament light chain. Zhonghua Yi Xue Yi Chuan Xue Za Zhi 28:496-500
